# Linking morphology, performance, and habitat utilization: adaptation across biologically relevant ‘levels’ in tamarins

**DOI:** 10.1186/s12862-023-02193-z

**Published:** 2024-02-14

**Authors:** Patricia Berles, Jan Wölfer, Fabio Alfieri, Léo Botton-Divet, Jean-Pascal Guéry, John A. Nyakatura

**Affiliations:** 1 AG Vergleichende Zoologie, Institut für Biologie, Humboldt-Universität zu Berlin, Philippstr. 12/13, 10115 Berlin, Germany; 2 Present Address: Institute of Ecology and Evolution, University of Bern, Bern, 3012 Switzerland; 3 Department of Earth Sciences, University of Cambridge, Cambridge, UK; 4 Museum für Naturkunde, Leibniz-Institut für Evolutions- und Biodiversitätsforschung, Berlin, Germany; 5 La Vallée des Singes, Romagne, 86700 France

**Keywords:** Biomechanics, Field study, Integrative biology, Leaping behavior, Limb bones, Locomotion

## Abstract

**Background:**

Biological adaptation manifests itself at the interface of different biologically relevant ‘levels’, such as ecology, performance, and morphology. Integrated studies at this interface are scarce due to practical difficulties in study design. We present a multilevel analysis, in which we combine evidence from habitat utilization, leaping performance and limb bone morphology of four species of tamarins to elucidate correlations between these ‘levels’.

**Results:**

We conducted studies of leaping behavior in the field and in a naturalistic park and found significant differences in support use and leaping performance. *Leontocebus nigrifrons* leaps primarily on vertical, inflexible supports, with vertical body postures, and covers greater leaping distances on average. In contrast, *Saguinus midas* and *S. imperator* use vertical and horizontal supports for leaping with a relatively similar frequency. *S. mystax* is similar to *S. midas* and *S. imperator* in the use of supports, but covers greater leaping distances on average, which are nevertheless shorter than those of *L. nigrifrons*.

We assumed these differences to be reflected in the locomotor morphology, too, and compared various morphological features of the long bones of the limbs. According to our performance and habitat utilization data, we expected the long bone morphology of *L. nigrifrons* to reflect the largest potential for joint torque generation and stress resistance, because we assume longer leaps on vertical supports to exert larger forces on the bones. For *S. mystax*, based on our performance data, we expected the potential for torque generation to be intermediate between *L. nigrifrons* and the other two *Saguinus* species. Surprisingly, we found *S. midas* and *S. imperator* having relatively more robust morphological structures as well as relatively larger muscle in-levers, and thus appearing better adapted to the stresses involved in leaping than the other two.

**Conclusion:**

This study demonstrates the complex ways in which behavioral and morphological ‘levels’ map onto each other, cautioning against oversimplification of ecological profiles when using large interspecific eco-morphological studies to make adaptive evolutionary inferences.

**Supplementary Information:**

The online version contains supplementary material available at 10.1186/s12862-023-02193-z [108].

## Background

Each habitat confronts the animals living in it with functional demands, which over time might ultimately drive morphological adaptations. To gain insight into such eco-morphological adaptations, integrated studies that include analyses of both the organisms’ morphology and *in-vivo* performance in controlled laboratory environments and in the field are indispensable to determine the intricate interplay between the biologically relevant ‘levels’ of morphology, performance, and (behavioral) ecology [1–4]. The musculoskeletal apparatus of vertebrates constitutes an insightful study system for such research avenues because, first, the locomotor behavior and performance are comparably straightforward to measure in the field and the laboratory, and then this information can be used to derive and experimentally test biomechanical hypotheses of structure-function relations on the reasoning of Newtonian mechanics. Many studies thus far have focused on the relation between structure and function in terms of the mechanical properties of morphological characteristics (e.g., [5, 6]) and related these to coarse categories of the animals’ behavioral ecologies (e.g., [7–9]). Far fewer studies aspired to investigate the fundamental link between structure on the one hand and in-depth aspects of the behavioral profile, the habitat utilization, as well as the animal performance in the field on the other hand (e.g., [10–15]). This can be likely related to the fact that such in-depth observations of animal behavior in the natural habitats are difficult to obtain without disturbance of the animals and relies on well-habituated study groups necessitating research infrastructure such as field stations. Moreover, aiming to employ the valuable tools of the comparative approach, studying more than one species, requires dealing with time-consuming interspecific data collection. We here present such an in-depth case study on tamarins, a group of callitrichid primates with apparently very conservative anatomy [16], focusing on four closely-related species to interrelate variability in habitat utilization, leaping behavior and anatomy of the locomotor system.

Tamarins (belonging to Callitrichidae, within platyrrhine primates), as all other arboreal mammals, are faced with specific functional demands for the locomotor system due to discontinuous, narrow, and flexible supports, which these animals need to be able to navigate to bridge gaps and reach food sources such as fruits, flowers, and invertebrates (see recent review [17]). For example, the movement on thin and flexible (i.e., precipitously bending) terminal branches, where usually the fruit and flowers are located, represents a challenge for the required balance [17–21]. Accordingly, support diameter and support flexibility should be considered as environmental variables that exert significant selective pressure on the locomotor apparatus (e.g., to stabilize the shoulder and elbow on flexible supports [16]) of such arboreal mammals [22, 23]. Similarly, the ability to leap from one support to the next has often been interpreted as an adaptation to an arboreal lifestyle (e.g., [18]). In this context, also lianas, i.e., thin and flexible, vertical supports, were discussed for their potentially crucial role in the evolution of locomotor adaptations in primates [24].

Several tamarin species live sympatrically in the Amazon basin [25]. By forming “mixed-species groups’’, some tamarins are even syntopic, traveling and foraging together albeit in different forest layers [26–29]. Because of species-specific preferences for foraging and traveling in different microhabitats, differential leaping behavior has been documented among callitrichid species, too (e.g., [30–35]). This is also the case for two of the focal species of our study. The first, *Saguinus mystax*, travels primarily in the upper layers of the forest (79% of the time [26]) and uses mostly horizontal supports thinner than 10 cm during locomotion [34], whereas the second, *Leontocebus nigrifrons*, primarily uses vertical supports of larger diameter in the lower forest layers (87% of the time) [26, 34–38]. For both species studied by Berles and colleagues [26], it was shown that there was a preference for one leaping type regardless of the available supports in the different forest layers. *L. nigrifrons* mainly leaps from trunk-to-trunk, a leaping style that is observed in strepsirrhine primates too, although with some differences [39] (i.e. tamarins exhibit pauses for clinging and scanning the environment and land forelimbs first, whereas strepsirrhines show a rapid sequence of leaps, landing with the hindlimbs first; [32, 40, 41]). On the other hand, *S. mystax* primarily performs horizontal leaps between terminal branches [26]. The third species of this study, *S. midas*, lives primarily in the lower to upper forest layer and moves along medium-sized supports (2-10 cm) [42–45], suggesting similar behavior to *S. mystax*. In contrast, *S. imperator* (the fourth focal species of our study), similar to *L. nigrifrons*, moves mostly in the lower forest layers but, unlike *L. nigrifrons*, predominantly uses smaller oblique supports for locomotion [37, 46].

**Fig. 1:**
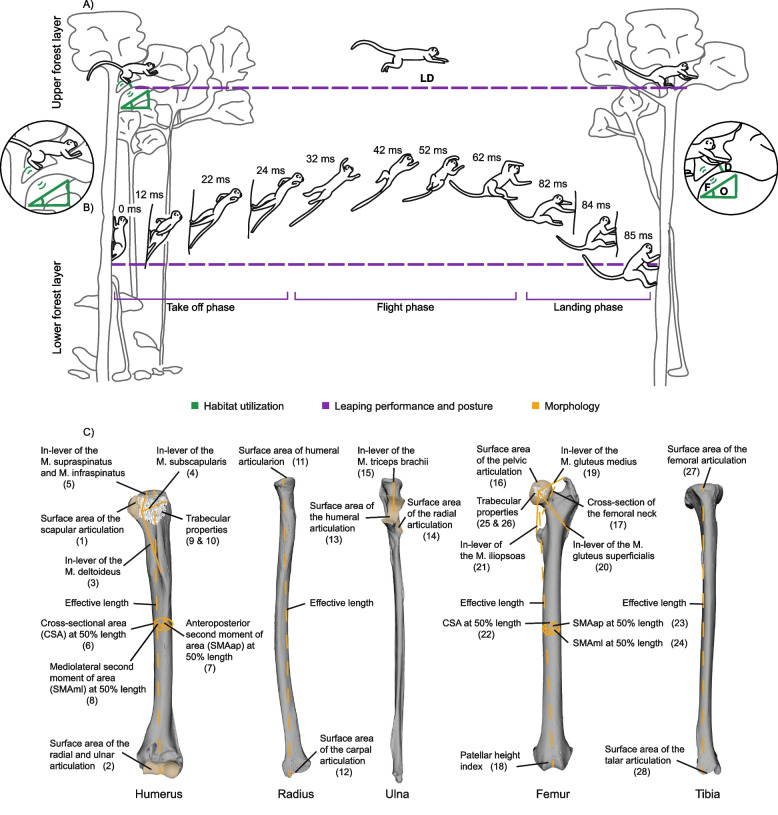
Schematic illustration of data acquisition. **A** Characterization of habitat utilization. Habitat characteristics and posture were recorded in the field/park and are shown in green (diameter, D; orientation, O; flexibility, F). **B** Leaping performance and posture. Horizontal leaping distance (LD) was recorded in the field/park, time-related performance measures were extracted from camera recordings and an exemplary trunk-to-trunk leap of *L. nigrifrons* with a leaping distance of 1.5 m is highlighted in violet (take-off-, flight-, and landing-phases in ms). **C** Acquired morphological variables. Measurements on the bones are highlighted in orange. Anterior view of the left humerus, ulna, radius, femur, and tibia of the specimen *S. mystax* AMNH 188,178. The numbers in panel C refer to the labelled data points in Fig. 2C. See text for more information

In the upper forest layers, crossing gaps usually involves leaps out of balancing quadrupedal movement on terminal branches [35, 41]. Due to the lack of horizontal supports in the lower forest layer, primates must use trunk-to-trunk leaps from a vertical clinging position to cross gaps between supports (Fig. 1) [47, 48]. Four types of leaps can be distinguished in tamarins. The first type represents long acrobatic downward leaps in horizontal body posture, in which tamarins cross horizontal distances of more than 5 m in the upper canopy. Typically, such leaps begin and end on thin, flexible, terminal branches [31, 33, 40, 41]. Sometimes several small twigs are grasped by one hand or foot at the same time. A second type of leaps in horizontal body posture is known as a bounding leap. This short leap involves a quadrupedal locomotor sequence with longer airborne phase on oblique and horizontal branches and depends on powerful limb extension to achieve the similar heights during take-off and landing [33, 40]. A third type of leaping in tamarins is a stationary leap, in which animals hold a stable posture on inflexible support of various angles of inclination before leaping [41]. As a fourth type, trunk-to-trunk leaps (Fig. S1) are specific in that the animals always start and land in a fully crouched and static vertical head-up posture on vertical supports [41]. These trunk-to-trunk leaps require several mechanical adaptations of the limbs. In addition to generating sufficient impulse during take-off through a powerful extension of the hindlimbs to bridge the horizontal distance, the body must be rotated during the flight phase [49] and the forelimbs, which are usually extended during the flight, must be able to withstand the compressive forces during landing [40, 41, 50, 51].

Previous studies on differences in morphology in tamarins, indicate a strong relationship between species’ morphological variation and already observed ecological variability within mixed-species groups [52–54]. For example, *L. nigrifrons*, which prefers trunk-to-trunk leaps, shows greater individual variation in morphology than *S. mystax*, which is related to greater variability in postural behavior found in field studies [54]. Also, *L. nigrifrons* exhibits some osteological features in the knee (e.g. a longer patellar groove, shorter articular facet on the patella and longer femoral condyles) that may be related to trunk-to-trunk leaps, as suggested by Garber and Davis [54], due to the strong flexion followed by extension during take-off. The forelimbs appear more gracile, e.g., due to a narrowing of the humeral bi-epiphyseal width, medial epicondylar width, and anterior and posterior trochlear width [55]. In a recent clade-wide comparative study by Botton-Divet and Nyakatura [16], the authors analyzed a variety of limb long bones and found some notable anatomical differences between the trunk-to-trunk leapers and the horizontal leapers within the callitrichids. For example, the hindlimbs of the trunk-to-trunk leapers were shown to have a proportionally smaller femoral head and a larger lesser trochanter, whereas the horizontal leapers have a more expanded trochlea at the humerus, which could provide greater stability of the elbow [16]. However, the study by Botton-Divet and Nyakatura [16] does not take into account the different ecological context, like support use and leaping performance, faced by the species under study.

Taking all of this into account, the diversity of habitat characteristics in these arboreal mammals allows for a detailed correlation between habitat utilization, performance, and morphology. Since the monophyletic taxon of tamarins is only ~ 14ma old [56] and since tamarins have very similar body sizes [36], morphological differences are likely reflective of adaptations to the specific functional demands resulting from their differing ecology [16]. In this study, we first provide information on behavioral data of leaping of four species of tamarins. We quantify habitat utilization in terms of support dimensions, orientation, and flexibility. Leaping performance was characterized in terms of leap distance and duration of sub-phases of leaps, also accounting for postural differences. While the leaping performance influences the magnitude of exerted forces, posture during take-off and landing determines the predominant direction of the involved forces. We here define performance from a strictly biomechanical viewpoint as an observed locomotor trait [15] and thus describe a habitual load caused by leaping, and not, as often used in the literature, the maximum performance of an individual, which occurs rather rarely in the natural habitat [57]. This is based on the experimental observation that the habitual load causes a dynamic change of bone structure [58–60]. Since habitual loads are difficult to quantify in the field, we rely on kinematic parameters which reflect the forces involved during leaping to characterize these loads. As proxies, we use the leaping distance and the temporal subphases of the leaps. The duration of the take-off subphase was demonstrated to be correlated to leaping performance, for example, in mouse lemurs [61]. By determining the duration of the take-off subphase we gain insight into the time available for leg extension, and thus, the impulse generated [62]. Similarly, the landing subphase informs on the duration of leg flexion. The leaping distance should be correlated to the peak support reaction forces exerted during leaping [63] while the relation between leaping distance and the duration of the flight subphase provides an idea of the jumping velocity. In addition, we measure various internal and external osteological features of the long bones from museum collection specimens of the four studied species. External features include muscle in-levers, robustness features, and limb proportions (Fig. 1C). Diaphyseal and epiphyseal internal structure were measured since they have been shown to reflect eco-morphological adaptation to locomotor biomechanical loadings in primates (e.g., [64–68]). The aim is to first find patterns in both habitat utilization and leaping performance that reflect distinct preferences of the tamarin species.

We have the following predictions for the level of habitat utilization:

1. *S. midas*, similar to what was already shown for *S. mystax* [26], predominantly uses horizontally oriented supports with a small diameter, due to the average height of stay in higher forest layers and the resulting availability of supports [69].
2. *S. imperator*, independent of the preferred lower forest layer [37], predominantly uses horizontal and oblique supports with small diameter [46].
3. *L. nigrifrons* is a specialist in leaping on large, vertical supports [26].

Based on this, we have the following predictions for the level of body posture and leaping performance:

4. The *Saguinus* species predominantly perform leaps in a horizontal body posture, while *L. nigrifrons* is a specialist for leaps in a vertical body posture independent of the available support type.
5. Vertical leaps cover a greater distance, which is also reflected in a longer duration of the flight subphase.
6. Greater leaps in general and horizontal leaps in particular [70] have longer durations of take-off and landing subphases.

Once species categorization is possible on these two behavioral ‘levels’, i.e., habitat utilization and leaping performance, we can hypothesize different functionally relevant demands on their locomotor morphology and test these on morphological data.

Based on the available literature, we have the following predictions for the level of locomotor morphology:

7. *L. nigrifrons* shows the strongest bone robustness as a result of the higher compressive stress on the forelimbs and hindlimbs during landing on large inflexible supports and relatively longer hindlimbs that benefit long leaps like in frogs [63] and galagos [71] instead of constant quadrupedal locomotion.
8. The *Saguinus* species have greater stability in the shoulder and hip joint for good balance on the flexible supports in addition to lower bone robustness [16] since horizontal leaps are mostly performed on flexible supports and the compressive stress during landing is lower.
9. Species with less variable habitat utilization display a smaller degree of trabecular anisotropy.

We believe that this study will not only contribute to a better understanding of the evolution and locomotor adaptations of tamarins, but also to a more general understanding of how eco-morphological adaptation manifests on different biological ‘levels’.

## Materials & methods

We collected data on three biologically relevant ‘levels’: (i) habitat utilization, (ii) leaping performance and posture, and (iii) morphology of the locomotor apparatus (Fig. 1). Data for habitat utilization and leaping performance were jointly collected in the wild and in a naturalistic park with different primates whereas data characterizing the locomotor morphology were collected from skeletal material of 12 museum collection specimens (three per species).

### Study sites and study groups

For this study, it was important to select groups of individuals whose natural behavior can be observed without disturbance by the presence of the observers. For this reason, we chose the following study sites. First, a field study was conducted during the dry season from June to October 2017 in the Amazon lowlands of north-eastern Peru at the “Estación Biológica Quebrada Blanco” (EBQB). The mean temperature during this period was 26.9 °C and the mean monthly precipitation was 175 mm/m² (Tamshiyacu, Peru; data from https://www.accuweather.com/de/pe/tamshiyacu). The station is located at 4°21’S and 73°09’W, on the right bank of the Quebrada Blanco, a small tributary of the Rio Tahuayo that empties into the Amazon (more detail and a figure showing the location of the station can be found in Berles et al. [26]; Heymann and Tirado Herrera [72]). The primary forest at EBQB consists predominantly of a dense canopy at a height of 25-30 m [73]. A mixed species group consisting of six adults and two juvenile individuals of *S. mystax* and three adult individuals of *L. nigrifrons* was studied. But we collected data on adult individuals only. The sex of the animals was neglected in this study. The tamarins of EBQB are well habituated to the presence of human observers. The activity data of the mixed-species group were recorded for a total of 68 days in the field. In total, *S. mystax* was observed for 586 h and *L. nigrifrons* for 519 h (mostly parallel to *S. mystax*).

A second behavioral study was conducted in October 2018 in Romagne, France, in a park hosting different primate species (“La vallée des singes” hereafter referred to as park study in contrast to the field study at the EBQB). The mean temperature during this period was 14 °C and the mean monthly precipitation was 49.6 mm/m² (Poitiers, France; data from https://www.wetterkontor.de/de/wetter/europa/extremwerte-frankreich.asp). The park consists of artificial islands overgrown with large trees and bushes with ropes between the trees to provide additional connections. The tamarins have the option to reside in temperature-controlled houses during the nights and winter months. The islands are separated by wide moats to prevent the monkeys from escaping. On these islands, visitors can explore the habitat the monkeys live in, so the tamarins are very used to observers. Here, the subjects of the study were three individuals of *S. midas* on one island and five *S. imperator* on another island. The activity data of the tamarins in the park were recorded over 14 days. *S. midas* was observed for a total of 21.5 h and *S. imperator* for a total of 23.5 h. Each data set was collected by the first author. Altogether, 5920 leaps were observed, 2347 leaps for *L. nigrifrons*, 2730 leaps for *S. mystax*, 359 leaps for *S. imperator* and 484 leaps for *S. midas*.

### Determination of habitat characteristics

The support properties of the home range of the study-site in Peru have been described in Berles et al. [26] and will be used here. In order to characterize the structure of the forest of the two islands in “La vallée des singes’’ and to determine the spectrum of available supports [74], one Whittaker plot for the island of each species was created (Table S1). The procedure was done identically at both study sites.

### Behavioral data acquisition

**Fig. 2:**
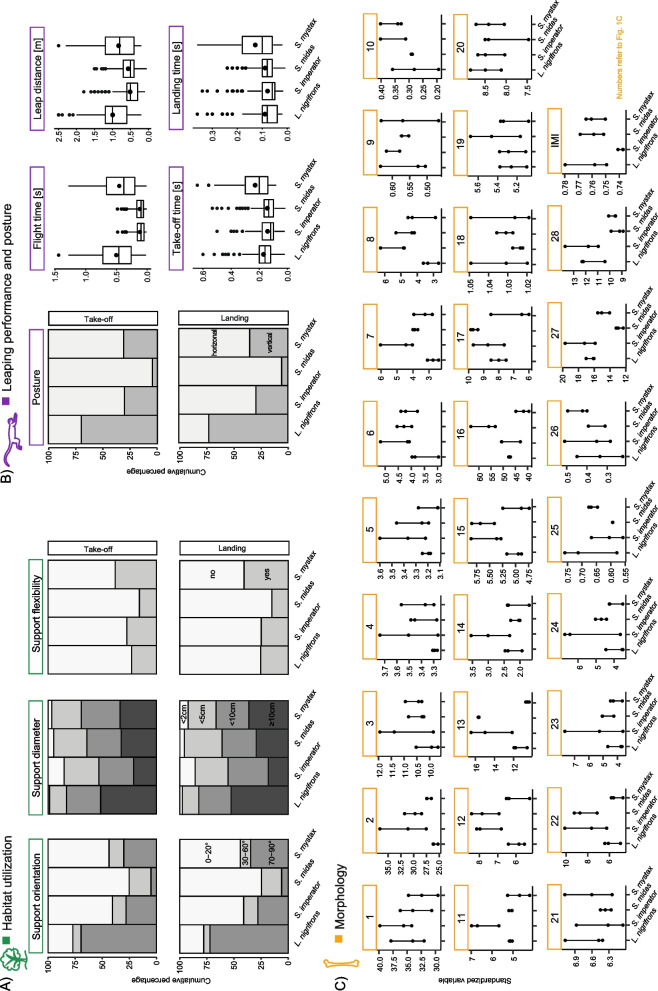
Data split by species. **A** Habitat utilization, (**B**) Leaping performance, (**C**) Morphology (standardized variables, compare to Fig. 1C). Humerus: (1) surface area of the scapular articulation, (2) surface area of the radial and ulnar articulation, (3) in-lever of the M. deltoideus, (4) in-lever of the M. subscapularis, (5) in-lever of the M. supraspinatus and M. infraspinatus, (6) cross-sectional-area (CSA) at 50% length, (7) anteroposterior second moment of area (SMAap) at 50%, (8) mediolateral second moment of area (SMAml) at 50% length, (9) trabecular degree of anisotropy (DA), (10) trabecular bone volume fraction (BV.TV); Radius: (11) Surface area of the humeral articulation, (12) Surface area of the carpal articulation; Ulna: (13) surface area of the humeral articulation, (14) surface area of the radial articulation, (15) in-lever of the M. triceps brachii; Femur: (16) surface area of the pelvic articulation, (17) Cross-section of the femoral neck, (18) patellar height index (patellar width projected onto surface/patellar width), (19) in-lever of the M. gluteus medius, (20) in-lever of the M. gluteus superficialis, (21) in-lever of the M. iliopsoas, (22) CSA at 50% length, (23) SMAap at 50% length, (24) SMAml at 50% length, (25) DA, (26) BV.TV; Tibia: (27) surface area of the femoral articulation, (28) surface area of the talar articulation. Percentages of utilized categories of the studied support characteristics and posture can be found in numerical form in supporting information Table S2

In the field study, the horizontal distance covered during leaping, the flexibility, orientation and diameter of the support as well as the posture of the animal at take-off and landing were visually estimated and recorded into a protocol (Fig. 1 in green, raw data can be found in Fig. 2). For this, every 30 min, the first visible individual was followed and all behavioral data were noted as long as it remained visible. For each leap that was observed, all characteristics were noted [26]. In addition, during the rest of the time, each visible leap of all individuals, with all associated parameters, was noted. In the park study, all support properties with a support height ≤ 2 m were measured and otherwise estimated as in the field study. Since visual estimation is essential for our study, we had all data recorded by only one trained observer, in our case the first author, as suggested by Bezanson and colleagues [75]. The first author in our study trained the visual estimation of support inclination, tree height, and distances previously both in Germany, in the Steigerwald, a full-leaved foliage forest in central Germany, and in a one-month pilot study directly in the rainforest [26]. In addition, the leaping behavior of the four species was recorded with a camcorder (Panasonic VW-ACT190, 50fps) in order to evaluate the durations of sub-phases of the individual leaps later in the lab (see below and violet boxes in Fig. 1, raw data see Fig. 2). These videos were acquired for all leaps observed in the park study, but only for a subset of all leaps observed in the field, since it was logistically impossible to film all the documented leaps at the same time. The field/park study notes were then correlated with the videos using the time code of the videos and a written protocol. Using the camera recordings, each leap was divided into a take-off phase, a flight phase, and a landing phase (violet boxes Fig. 1). The take-off phase starts with the first observed loss of contact of any one of the four limbs and ends with the observed loss of contact of the last of the four limbs. This is followed by the actual flight phase. The flight phase ends with the first contact of any limb with the new support. The subsequent landing phase ends with the last of the four limbs contacting the new support. Using high-fps video recordings we measured the duration in milliseconds of each phase. The time resolution was 20 ms.

In summary, variables characterizing the ‘level’ of habitat exploitation were support orientation, support diameter, and flexibility during take-offs and landings, respectively. The habitat variables were of categorical nature. We used three orientation categories (0–20°, 30–60°, 70–90°), four diameter categories (< 2 cm, <5 cm, < 10 cm, ≥ 10 cm), and two flexibility categories (yes, no). The ‘level’ of leaping performance was characterized by the horizontal distance of leaps as well as the posture during take-off and landing, respectively, and the duration of take-off-, flight-, and landing phases. The performance variables were of mixed categorical (posture) and continuous (distances and durations) nature. Posture (i.e., the body position during leaping) included two categories that were noted regardless of the support used: typical trunk-to-trunk leaping posture (i.e., monkeys clinging to the supports in a vertical position) and typical horizontal leaping posture (i.e., monkeys were in a pronograde, quadrupedal body position) (Fig. 1).

### Bone data acquisition

Bones were obtained from the Field Museum of Chicago (FMNH) and the American Museum of Natural History in New York (AMNH). The specimens from the FMNH were CT-scanned at the PaleoCT Scanner Facility of the University of Chicago using a GE phoenix V|tome|x. Specimens from the AMNH were CT-scanned at the Shared Materials Instrumentation Facility (SMIF) of Duke University. The resolution of the scans varied from 15.5 to 18 μm. As all bones of a single specimen were scanned as a batch, single bones were then cropped using Fiji [76] and CT scans were segmented using Amira 6.0.0 (Thermo Fisher Scientific). All meshes are available on demand from Morphosource (see specimen details in Table S3). Importantly, the epiphyses of two humeri and one femur were not completely fused, meaning that the specimens were likely subadult (humeri: *S. imperator* FMNH 98,035 and *S. midas* FMNH 93,236 femur: *S. midas* FMNH 93,236).

### Quantification of the bone internal structure

Diaphyseal and epiphyseal internal structure (cross-sectional properties [CSP] and trabecular architectural properties, respectively) were quantified in the humeri and femora following part of the procedure in Alfieri et al. [77] (Fig. 1 in orange, for raw data see Fig. 2). The two bones were oriented in anatomical standard position following Ruff [66] using VG Studio Max 3.3 (Volume Graphics, Heidelberg, Germany). Oriented bones, exported as image TIFF stacks, were then imported to Fiji for internal structure quantification. Regarding diaphyseal properties, we quantified the cross-sectional area as well as the anteroposterior and mediolateral second moment of area at 50% bone length. Regarding epiphyseal properties, we included the trabecular degree of anisotropy (DA) and the trabecular bone volume fraction (BV.TV). Further details on CSP and trabecular properties and the procedures employed to quantify them are included in Supporting information note 1.

### Quantification of external bone morphology

The segmented CT scans were exported as a surface mesh from Amira. Measurements of the outer morphology of the humerus, ulna, radius, femur, and tibia were obtained on these meshes in Geomagic Wrap (3D Systems 2017). We measured the effective length of the humerus, radius, femur, and tibia to calculate the intermembral index (IMI: (humeral length + radial length) / (femoral length + tibial length)). A low IMI indicates relatively longer hindlimbs and hence, a specialization for vertical leaping [78]. We further measured robustness variables that inform on the potential to resist stresses as well as the length of muscle in-levers that inform on the potential to generate and absorb joint torques. A detailed description and depiction of the measurements are provided in the supporting information (Figs. S2, S3, S4, S5, S6) and a brief overview of all measurements is displayed in Fig. 1. All internal and external morphological variables were continuous and we size-corrected them prior to further analysis using the centroid sizes of the humerus and femur (see Supporting information note 2 for details).

### Missing data imputation and sampling bias correction

All subsequent analyses were conducted in R Version 4.2.2 [79]. Measurement values were missing for the performance variables due to take-off and landing events being out of sight during recording. In total, 8.5% of the recorded leaps had at least one missing subphase. The take-off was missing in 1.7% of the recordings, the flight subphase in 7.2% and the landing subphase in 6.7%. Also, data were missing in the trabecular variables due to unfused epiphyses in two humeri and one femur. The missing data were imputed for both data sets separately using the R package missMDA [80]. See Supporting information note 3 for more details. We imputed the data after sampling bias correction (see below) because the frequency of missing data was linked to the support use and height of the leaps. Specifically, leaps high up in the canopy had missing data and removing them before bias correction would have increased the existing bias towards including disproportionately more vertical leaps from the lower forest layers. Hence, we decided to rather rely on imputed data for these leaps instead of increasing the disbalance of the recorded leaps even more.

### Sampling bias correction for the performance data

As most of the video footage used to capture durations could only be recorded in the lower layer of the forest, the ratio of horizontal to vertical leaps was likely biased toward vertical leaps. This was particularly likely for the two species that were filmed in the wild where trees were higher (Table S1). Thus, the data set had to be adjusted to correct for this bias. This procedure resulted in a selection of 1092 of the 1271 observations (Table S4C). See Supporting information note 4 for more details. A summary of the number of observations per species and per ‘level’ after each step (data collection, missing data imputation, and sample bias correction) can be found in Table S5.

### Cluster analysis and dimensionality reduction

The R packages FactoMineR [81] and clValid [82] were used for data ordination, cluster analysis, and cluster validation/assessment, respectively. As mentioned above, we first used cluster analysis on the ‘levels’ of habitat utilization and leaping performance separately to explore whether the four studied species can be categorized into groups that reflect a dichotomy between trunk-to-trunk-leaping and horizontal leaping. Each cluster analysis was preceded by an ordination analysis, but all ordination dimensions were used for clustering. The ordination was necessary for the datasets concerning the habitat utilization and leaping performance to transform the categorical variables into pseudo-continuous ordination scores from which distance metrics could be computed. We also used the first two ordination dimensions to visualize the mapping of clusters between the different ‘levels’, justifying also the ordination of morphological variables. For habitat utilization, which consisted of categorical variables only, we used multiple correspondence analysis (MCA; MCA function from FactoMineR), which is equivalent to principal component analysis (PCA) for quantitative data. For simplicity, we refer below to principal components (PCs) to the dimensions of all dimensionality reduction techniques. In MCA, variables are split into their categories which are in turn transformed into continuous variables, giving larger weight in the generation of PCs to categories shared by fewer individuals. On the adjusted data set of the performance variables, we performed a factor analysis of mixed data (FAMD; FAMD function from FactoMineR). This analysis includes both categorical and continuous variables. On the morphological variables, we applied a PCA (PCA function from FactoMineR).

Hierarchical clustering was conducted each time on the PCs using the HCPC function from FactoMineR. The algorithm operates by assigning each observation to a cluster and merging them to higher ‘level’ clusters step by step by combining the two clusters at a time that share the closest Euclidean distance in the multidimensional space spanned by the PCs. The WARD method was used to avoid chain effects that can complicate cluster generation in the presence of coalescing clusters. The clusters were then selected by cutting the cluster tree and a k-means consolidation was used post hoc to consolidate clustering. We decided upon the most meaningful clustering primarily by using inertia gain (i.e., how much variance can be additionally explained by adding another cluster) as provided by the HCPC function. Different cluster determination criteria exist and, hence, the resulting interpretations might depend on the favored criterion. Although we decided to determine the number of clusters via the criterion of inertia gain, we additionally assessed the reliability of different numbers of clusters using three more indices. These are called connectivity [83], Dunn index [84], and silhouette width [85]. The measure of connectivity indicates how well similar observations are clustered together as determined by the ‘k-nearest neighbors’ method. It can range from zero to infinity and should be minimized for good clustering. The Dunn index is a ratio of the smallest distance between the clusters to the largest intra-cluster distance. It falls between zero and positive infinity and the larger the value, the better the cluster discrimination. The silhouette width is the average of the silhouette values among observations. The silhouette value measures the degree of confidence that an observation is assigned to a particular cluster. The value falls between − 1 and + 1 with poorly clustered observations being close to -1 and well-clustered observations being close to + 1.

We cut the tree into two to six clusters for these analyses, each time computing the measures of clustering quality using the package clValid [82]. Two to six clusters were chosen since two clusters were expected regarding the “trunk-to-trunk leaper vs. horizontal leaper” dichotomy, three clusters seemed plausible with an additional intermediate or more generalist locomotor behavior, four clusters could represent the four species, and five to six clusters would indicate that no interpretable clustering could be achieved. Testing a larger number of clusters did not appear insightful to us.

After selecting a clustering for each level, we used the five data points closest to the respective cluster centroid to characterize habitat utilization and performance clusters. To ensure an accessible overview of the characteristics of the morphological clusters, we created bar plots of differences between the mean values of clusters instead. We calculated the relative frequency of each species falling into the respective cluster and used a χ²-test to evaluate whether there is a significant association between the clusters and the species within each ‘level’. We further generated graphs with the first two principal components of each ‘level’ and visualized the loadings of the original variables onto these to illustrate and explore clustering trends. However, care must be taken in interpretation in case the loadings of the original variables onto these 2D subspaces are low, which would indicate a poor representation of these variables in these two dimensions. The loading between a continuous variable and a PC was defined as their Pearson’s correlation coefficient and the loading between a categorical variable and a PC was defined as the R² obtained from an ANOVA analysis.

### Inferential statistics

Cluster analysis and dimensionality reduction provide insight into the major trends between species within each biological level that we studied. However, we also plotted the raw data of each level to find patterns which might not be captured by these analyses. For this purpose, we used inferential statistics to compare differences between the four species in each trait of the levels of habitat utilization and leaping performance and posture. We refrained from conducting inferential statistics with the morphological traits because of the small intraspecific sample sizes (see Supporting information note 5 for more details).

### Specific challenges of linking different 'levels'

For the habitat characterization it would be optimal to have a detailed overview about all available supports within the home range of the monkeys. Unfortunately, it was not possible to determine exact proportions of available supports for each leap. Nevertheless, based on the similar trends observed in other studies, we believe that our estimates and measurements reflect a good pattern of support use during leaping by the four species.

In theory, it would be ideal to have more than four species from which the three levels are extracted. With four species, we only have four degrees of freedom at maximum for a statistical analysis of adaptation. Additionally, it would be optimal to study at least one species in both field and park, to provide a valuable means for validating and comparing methods and observations. Unfortunately, this was not possible, due to the rather rare occurrence of the species in naturalistic parks.

Also, the individuals studied in the field are not the same from which we obtained the morphological data, limiting us to the comparison of species’ means. In the optimal case, data from all the ‘levels’ here analyzed would be obtained from the same individual. However, no animals were sacrificed for this study. These limitations prevented us from conducting any reliable inferential statistical analysis across the levels. Thus, we decided to keep this study within an exploratory framework using mostly descriptive statistical methods.

## Results

### Habitat utilization

**Fig. 3:**
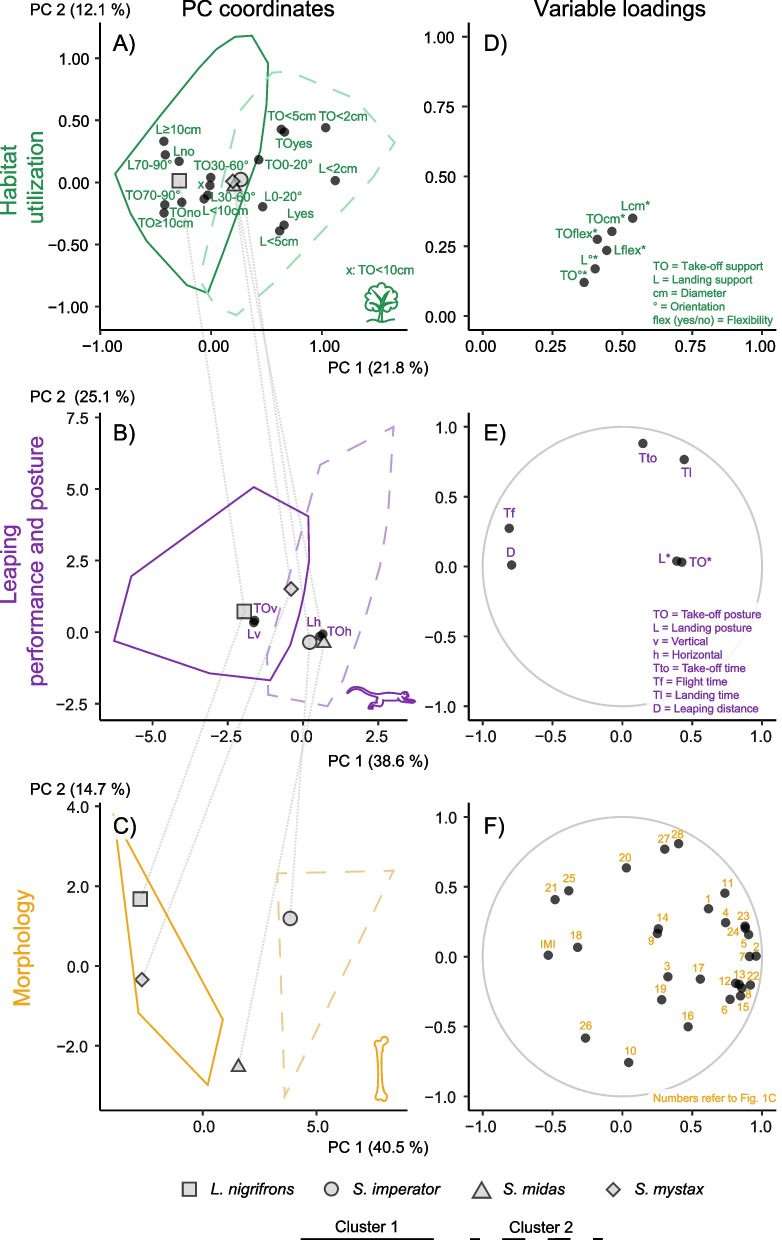
Principal component graphs of biologically relevant ‘levels’. The planes spanned by the first two principal components (PCs) from the habitat utilization (**A**), leaping performance and posture (**B**) and morphology (**C**) datasets are shown with grey symbols representing the mean values of the species and black symbols representing the mean values of the categorical variables. The grey lines connect the species means to highlight cross-level differences. Variable loadings of the corresponding PC analysis (**D**, **E**, **F**) are found right to the PC graph. Loadings of continuous variables represent Pearson’s correlation coefficients and can range from − 1 to + 1. The closer a point falls to the circle’s margin the better is its variable’s representation in this 2D subspace (falling on the margin indicates total representation). Loadings of categorical variables (indicated by asterisks) represent R² values from an ANOVA and can range from 0 to + 1. The point labels in panel F correspond to the numbered morphological variables in Fig. 1C (the point for IMI referring to the effective length measurements used to compute the index)

The inertia gain criterion suggested two clusters of support use for the leaps of all four species (Fig. 3A, Fig. S7). On the contrary, the measures used for cluster validation suggest the selection of six instead of two clusters (Table S6), indicating that the two clusters were poorly separated. However, only the connectivity was strongly improved (187.73 for two clusters and 29.3 for six clusters), whereas the Dunn index (0.22 for two clusters and 0.28 for six clusters) and the silhouette width (0.21 for two clusters and 0.26 for six clusters) improved marginally. We thus decided to use the two clusters from the inertia gain criterion for further interpretation, because they reflect a weak, but meaningful trend in the support use variability across species.

According to the five most typical leaps, cluster 1 was characterized by vertical (70–90°), thick (> 10 cm), and inflexible supports, for both take-off and landing, which can be considered trunk-like supports. Cluster 2 on the contrary was characterized by horizontal (0–20°), thin (< 5 cm), and flexible supports (Table S7), which can be considered canopy-like supports. We found a significant association between clusters and species (χ²(df = 3) = 357.77, *p* < 0.001) that is majorly driven by *L. nigrifrons* (Fig. 3A). In particular, 81% of the observed leaps of *L. nigrifrons* fell into cluster 1, whereas *S. mystax*, *S. midas* and *S. imperator* were very similar with 54–60% of leaps falling into cluster 1. Accordingly, *L. nigrifrons* can be considered rather specialized in the exploitation of trunk-like supports, whereas the three *Saguinus* species can be characterized as more generalist (Fig. 3A, D). The three *Saguinus* species, despite being all generalist, differed significantly in most of the support characteristics (Table S8), which is not captured by the cluster analysis. In particular, *S. midas* used flexible supports and supports with 0–20° orientation more frequently than the other two species (Fig. 2). *S. imperator*, on the other hand, used the support < 2 cm more and those ≥ 10 cm less frequently compared to the other two *Saguinus* species. *S. mystax*, at last, stood out in using flexible supports more frequently than the other two species (Fig. 2).

The described cluster trends are well-captured by the first two principal components, despite them only explaining 34% of the variance (Fig. 3A, Fig. S8). PC1 separates the two clusters and all variables load strongest on PC1 compared to PC2 (support diameters are represented the best and support orientations the worst). Also, the centroid of *L. nigrifrons* is positioned on the far left of cluster 1 and the centroids of other three species closely together near the intersection of both clusters. This in addition to the cluster results described above render the graphs reliable to illustrate the clustering trend of leaps and its association with the species. However, the poor low-dimensional representation of support use variability, the continuous scatter of data points in the space spanned by the two first PCs and the poor clustering are all indicators of a low correlation among habitat utilization variables (i.e., the leaps were characterized by a large diversity of combinations of support characteristics).

### Leaping performance and posture

The inertia gain criterion of the cluster analysis again suggested two clusters also for leaping performance and posture (Fig. 3B, Fig. S7). However, the three measures used for cluster validation suggest the selection of different numbers of clusters (Table S6). The connectivity supported the choice of two clusters, whereas the Dunn index suggested five clusters and the silhouette width three clusters instead of two. Nevertheless, both latter indices were only slightly improved by increasing the number of clusters, with the Dunn index increasing from 0.03 to 0.05 and the silhouette width increasing from 0.367 to 0.372. Hence, we chose the two clusters supported by the inertia gain criterion and the connectivity index for further interpretation.

Cluster 1 is characterized by a vertical body posture during take-off and landing (though one of the five data points closest to the centroid was a horizontal-to-horizontal leap; Table S7). It is further characterized by relatively longer leaping distances and flight durations and by short take-off and landing durations. Cluster 2 is represented by a horizontal body posture during both take-off and landing, as well as a shorter horizontal leap distance and a shorter flight time on the one hand and longer take-off and landing times on the other hand (Table S7). We found a significant association between the clusters and the species (χ²(df = 3) = 425.88, *p* < 0.001) with large differences among all species. In particular, when looking at the percentage of leaps of each species falling into the first cluster in descending order, it was 86% for *L. nigrifrons*, 66% for *S. mystax*, 20% for *S. imperator*, and 3% for *S. midas*. Thus, *S. midas* and *S. imperator* can be considered specialized in short leaps with horizontal postures, *L. nigrifrons* specialized in long leaps with vertical take-off and landing postures, with *S. mystax* falling in between with a non-specialized leaping behavior. However, the cluster results do not reflect the fact that *S. midas* leaps significantly more frequently in a horizontal posture than the other two *Saguinus* species, that did not differ significantly in this regard (Fig. 2, Table S9).

The described cluster trends are mostly well-captured by the first two principal components, which explain 64% of the variance (Fig. 3B, Fig. S8). PC1 separates the two clusters, and body posture, flight duration and horizontal distance load stronger on PC1 compared to PC2. However, take-off and landing durations load stronger on PC2 and do not appear to contribute to the clustering. Using these variables for cluster discrimination as suggested by the five most typical leaps described above might thus be misleading. PC2 shows that *L. nigrifrons* and especially *S. mystax* display longer take-off and landing durations than the other two species (Fig. 3E). The distribution of species means with the close proximity of the centroids of *S. midas* and *S. imperator* reflect the three clusters supported by the silhouette width index above. All of this suggests that the categorization of the performance depends on the performance variables under consideration.

### Locomotor morphology

Based on the results in terms of habitat utilization and leaping performance, we amended our predictions for *S*. *mystax*. *S. mystax* is similar to *S. midas* and *S. imperator* in support use, but exhibits larger leap distances. Based on this, we expect *S. mystax* to have relatively more robust bones to withstand higher compressive stress than *S. midas* and *S. imperator*, but at the same time relatively less robust bones than *L. nigrifrons*. The detailed predictions for each of the internal and external bone morphological variables (Fig. 1C) can be found in Table S10.

According to the inertia gain criterion, the cluster analysis revealed two clusters of similar morphologies (Fig. 3C, Fig. S7). They are also supported by the connectivity (9.34) as well as the silhouette width (0.29). The Dunn index (0.99) favors six clusters, but only marginally improves compared to its value when choosing two clusters (0.6) (Table S6). A reason for this could be the small sample size. Consequently, we chose two clusters for interpretation.

Cluster 2 is characterized by larger values than cluster 1 in 24 out of 29 variables, although the degree of difference varies between about 0.25 to 2 standard deviations (Fig. S9). Cluster 1 is only characterized by larger values concerning patellar groove height, the length of the iliopsoas in-lever, IMI, as well as DA and BV.TV in the trabeculae of the femoral head. We found no significant association between the clusters and the species (χ²(df = 3) = 4.89, p = 0.18), which might be attributed to the small sample size. All three specimens of each *L. nigrifrons* and *S. mystax* as well as two *S. imperator* specimens and one *S. midas* specimen were assigned to the first cluster, the remaining three specimens falling into the second cluster. Thus, *L. nigrifrons* and *S. mystax* have less robust morphologies with reduced potential for joint torque generation whereas *S. imperator* and *S. midas* are more diverse in their morphology compared to *L. nigrifrons* and *S. mystax* with a trend toward increased robustness and heightened potential for joint torque generation.

The described cluster trends are well-captured by the first two principal components, which explain 55% of the variance (Fig. 3C, Fig. S8). PC1 separates the two clusters and 16 of the 29 variables have a loading above 0.5 on PC1 (Fig. 3F). *L. nigrifrons* and *S. mystax* are positioned in the first cluster which is associated with low PC1 scores, whereas *S. imperator* falls clearly into the second cluster associated with high PC1 scores. *S. midas* falls in between the two clusters. Six variables have a loading above 0.5 on PC2, but since none of the cluster validation indices suggested a third cluster that is separated along PC2 from the first two clusters, they might not be informative to explain the morphological variation between species.

## Discussion

The fundamental phenomenon of adaptation may only be understood by an analysis of the intricate relationship of the different biologically relevant ‘levels’ constituting it: from morphology to function, behavior, and environment [1–4]. Integrated studies of prey capture and locomotion in predators show that various performance characteristics, such as acceleration and deceleration, may depend on predator ecology [86–89], while factors such as habitat structure and locomotor performance [90–92], are reflected in morphological adaptations [12, 13]. Our study contributes to the understanding of the relationship between morphology, habitat utilization, and performance in the field. We collected data on habitat use and leaping behavior of the free-living mixed-species group *L. nigrifrons* and *S. mystax*, and of *S. midas* and *S. imperator* living in a naturalistic park, showing trends in support preference, body posture during leaping, and leap distance. Our results agree with the previously described great locomotor plasticity in tamarins in relation to their small body size spectrum [40], highlighting the importance of an in-depth analysis of support use and performance. Further, many of the morphological variables studied here contributed to a clustering of the studied species, but this clustering and its underlying morphological trends contradicted our predictions that were derived from the habitat utilization and performance analyses. These findings suggest a complex relationship between these three ‘levels’ of habitat use, performance, and morphology. We discuss the results of each ‘level’ in detail to highlight novel insights and discrepancies with other studies as well as potential connections between the three studied ‘levels’.

### Major differences in habitat utilization between *Leontocebus* and *Saguinus*

**Fig. 4:**
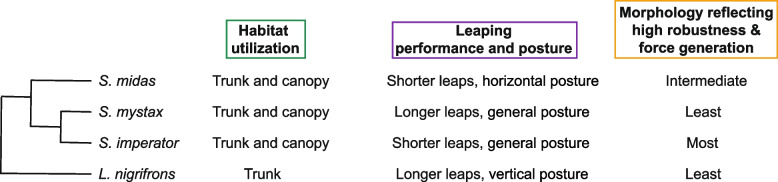
Cladogram of the four studied tamarin species with cluster characterizations for each of the three analyzed biologically relevant ‘levels’. The tree topology follows Botton-Divet & Nyakatura [16], but branch length information was omitted for simplicity

Support inclination, flexibility, and diameter are of great importance for arboreal primates, as they can vary within a single movement sequence, which constantly needs to be adjusted accordingly [93, 94]. We could show on the basis of the cluster results that the three *Saguinus* species can be described as generalists with a flexible choice of supports for leaping. *L. nigrifrons*, on the other hand, can be described as a trunk specialist in frequently using vertical (70–90°), thick (> 10 cm) and inflexible supports both during take-off and landing, as expected. This distinction between *Saguinus* as generalists and *L. nigrifrons* as a trunk specialist could indicate a phylogenetic signal in the habitat utilization pattern (Fig. 4). However, our results suggest that all studied tamarin species are in principle capable of coping with a variety of support use characteristics, as already described in the literature [26, 40, 41, 93]. Also, our results point out that the *Saguinus* species differ in how frequently they use certain support characteristics. The fact that *S. imperator* showed a stronger utilization of thin supports (also shown by Karantanis [93] and Buchanan-Smith [46]), but not particularly of more flexible and horizontally oriented ones compared to the other two, reflects the fact the *S. imperator* moves mostly on branches in the lower forest layers [37, 46]. *S. midas*, on the other hand, shows the most frequent utilization of horizontal supports as well as of inflexible supports. The latter fact contradicts previous studies that found that *S. midas* mostly leaps between terminal, flexible branches in the mid-forest layer [43, 44]. Perhaps this is related to the specific group of individuals under study or differences in habitat structure and indicates a larger intraspecific variability. *S. mystax*, on the other hand, stood out in using flexible supports most frequently. This is surprising, since this species also used vertical supports more frequently than the other two *Saguinus* species. Perhaps this reflects a more dedicated use of trunks from younger trees.

The only interspecific comparative studies besides ours [34] investigated the support uses of *S. mystax* and *L. nigrifrons*. The author could not find any differences in support sizes used for locomotion in these two species. Although *L. nigrifrons* used vertical trunks more often than *S. mystax*, both species rarely used supports larger than 10 cm in diameter [34]. In contrast, in our study, *L. nigrifrons* primarily used supports thicker than 10 cm in diameter for leaping (Fig. 2). This is surprising since we studied the primates at the same study site as Norconk [34] and might be related to intraspecific variability. Another explanation could be differences in the composition of the trees between subareas of the EBQB study site. However, mirroring our results, Norconk [34] found a clear difference in the support inclination used, with *L. nigrifrons* spending 50% of the observed time on vertical supports, while *S. mystax* most frequently used oblique and horizontal supports. In a study by Smith [38], *L. nigrifrons* used larger supports and vertical supports in more than 70% of the leaps, while *S. mystax* preferred thinner supports and used horizontal supports for leaping in more than 80%. In yet another study by Garber and Pruetz [33] at a site at the Rio Blanco, it was shown that *S. mystax*, used horizontal branches for locomotion twice as often as vertical ones, but small, medium and large supports were used relatively equally. In summary, the support uses of the four species here studied is mostly consistent with information in the literature, while differences between study groups might be related to intraspecific variability or differences in habitat structure. However, for example, in a comparative study of *S. mystax* at two different locations, it was shown that despite significant differences in forest structure, the overall pattern and frequency of movement and postural behavior hardly differed [33]. Also, in a mixed species group of *L. nigrifrons* and *S. mystax*, both species preferred their predominant support use regardless of the availability of supports in the different forest layers [26].

### Leaping performance partly corresponds to habitat utilization

We hypothesized that, based on the climbing height preferences reported in the literature, *S. midas* and *S. mystax* predominantly perform leaps in a horizontal body posture during take-off and landing. The distances between the branch-type supports are likely relatively short. This is why we also expected these species to leap short distances with short flight phases. On the other hand, we expected *L. nigrifrons* to start and land leaps in a vertical body posture and to leap the largest horizontal distance with the longest flight phase because of tree trunks being usually positioned further from each other than horizontal branches. We expected a more generalist leaping performance from *S. imperator* due to its preference for inclined supports with smaller diameters in the lower forest layers.

The predictions for *L. nigrifrons* were met, but the three *Saguinus* species showed intricate differences in their leaping performance that partly opposed our predictions in the case of *S. imperator* and *S. mystax*. According to our analysis, *S. midas* and *S. imperator* can be considered specialists for short horizontal leaps independent of the choice of support. This pattern is particularly evident for *S. midas* because it adopted a horizontal body posture during take-off and landing most frequently among the study species. Nevertheless, *S. midas* is known to be capable of long leaps when using vertical trunks and might leap as far as 7.6 m [95]. *S. mystax*, on the other hand, performed longer leaps than the other two *Saguinus* species in our study despite using a horizontal body posture as frequently as *S. imperator*. Perhaps, this can be explained by the use of specific support diameters, since *S. mystax* and *L. nigrifrons* both used the largest support diameters the most often and also leapt longer distances than the other two tamarin species. Nevertheless, *L. nigrifrons* covered the largest mean distance in our species sample with distances greater than 1 m making up 65% of the leaps (see Fig. S10). A similar observation was done in a study by Garber and colleagues [32], in which *L. nigrifrons* covered horizontal distances between 1 and 2 m in 51.5% of leaps, while only 9.5% were below 0.5 m and distances greater than 3 m accounted for only 2% of leaps. However, Garber and Leigh [41] found that *L. nigrifrons* covered a horizontal distance of less than 1 m during vertical leaps in 83% of the observed cases. Perhaps this is again related to the specific group of individuals under study as well as the respective habitat structure.

The habitat characteristics and performance cluster indices did not agree upon a single number of clusters, neither within each of these levels nor between both levels. This means that a straightforward relationship between both levels cannot be inferred. Instead, it appears that specific features of support use and leaping performance are linked to each other. For example, the fact that we found *S. mystax* to use flexible supports most frequently among the four species studied is consistent with it displaying the longest take-off and landing times. In contrast, the similarly long push-off durations during take-off in *L. nigrifrons* may be related to this species covering larger distances. Long take-off times are beneficial for generating large support reaction force impulses during push-off. This impulse may result in larger covered distances or may be lost for propulsion due to the bending of the flexible support [48]. In addition, it was shown for gibbons that this shift of the center of mass (CoM) further towards the landing support by increasing hip joint excursion during the take-off reduces the leaping distance [70]. This allows to compensate for the deflection of the support with minimal work for the CoM [70]. It would be interesting to study whether *S. mystax* and *L. nigrifrons*, when they travel together through the forest, choose supports with different flexibility properties while using supports with the same diameters. Furthermore, according to our results, the frequency of each posture adopted during take-off and landing follows the frequency of the support orientation (Fig. 2). In particular, vertical support orientations and vertical body postures are used with the same frequency within each species, and the same holds for horizontal supports and the horizontal body posture. The fact that this correspondence was observed in all four species suggests that all of them can adjust their body posture according to the support orientation faced at each leap.

### Morphology does not correlate to habitat utilization and leaping performance

Based on the results of the habitat utilization and the leaping performance of the studied tamarins, we expected the locomotor morphology of *L. nigrifrons* to reflect a larger potential to generate joint torques and resist forces associated with long distance leaps from and to inflexible supports compared to the *Saguinus* species. Only the lever-arm of the iliopsoas muscle was expected to reflect a larger potential for joint torque generation in *Saguinus*, because it would benefit stability while climbing on narrow supports. The IMI was expected to be smaller in *L. nigrifrons*, because relatively longer hind limbs compared to the other species are expected to contribute to the long-distance leaps performed by this species.

Although we found many morphological features to discriminate the studied species, none of these differences were according to our predictions. However, while we expected overall habitat utilization and locomotor performance to drive morphological adaptations, it might be that specific aspects of both could relate to the morphological similarity between *L. nigrifrons* and *S. mystax.* As discussed above, both these species were the ones that most frequently used the largest support diameters, and, more obviously, the ones that covered the larger leaping distances. Moreover, all the *L. nigrifrons* and *S. mystax* specimens were associated with cluster 1, which was characterized by larger values of DA and BV.TV on average. This is largely in agreement with expected functional adaptations of these two trabecular parameters. Concerning BV.TV, this trait is expected to positively relate to the magnitude of stresses acting on a joint (i.e., more bone volume in response to increased biomechanical stimulations, see Kivell [59] for a review). Hence, higher BV.TV in *L. nigrifrons* and *S. mystax* reflects a larger potential to resist biomechanical stresses experienced during long-distance leaps. A higher DA was previously detected in the femoral head of leaping strepsirrhines and related to their stereotypically oriented vertical clinging and leaping [96]. Perhaps, a more preferentially oriented direction of the trabeculae could also reflect the necessity to resist the large support reaction forces during take-off and landing. However, for all other studied traits it appears paradoxical that a lower potential for the generation of joint torque and resistance against forces is associated with larger leaping distances from comparably wider supports. Rather, leaping of tamarins from and to trunk-like supports should require the generation of large propulsive forces through a powerful extension of the hindlimbs during take-off and the withstanding of considerable compressive and bending forces acting on the forelimbs during landing [40, 97]. In case of *S. mystax*, it might be explained by its comparably frequent use of flexible supports. It is known that the peak take-off force is reduced by the flexibility of the support, which means that the animals have to generate higher forces during take-off from flexible supports than from rigid supports [48].

While we found *L. nigrifrons* and *S. mystax* to display a similar locomotor morphology with regard to skeletal measurements, Garber [40] found that *L. nigrifrons* has relatively longer arms, legs, hands and feet than *S. mystax*. This limb elongation was explained in connection with the preferred trunk-to-trunk leaping [40], since, as already mentioned, this type of leaping differs considerably from horizontal leaping due to a stationary, vertical holding position during take-off, which requires the animals to overcome substantial inertia. In addition, the body must be turned almost 180 degrees in flight. Figure S1 illustrates typical postures exhibited by the different species during trunk-to-trunk leaping during our study. Interestingly, only *L. nigrifrons* performs a strong rotation of the body axis during trunk-to-trunk leaps. The other three species also leap from a vertical holding position but are already facing the landing support with the flight phase being similar to that of a horizontal leap, where the body does not have to be rotated as much. Since the required muscle power is generated by the hindlimbs, long and/or well-muscled hindlimbs are advantageous [50]. Due to the notable dominance of the quadriceps femoris muscle in specialized strepsirrhine primates, the force from the knee and thigh can be transferred to adjacent joints and segments [98]. In our study, however, the gluteus medius muscle, based on its in-lever, appears to be suited for more powerful hip extension in *S. midas* and *S. imperator* than in *L. nigrifrons*. It is also difficult to relate the higher potential to generate joint torques and resist forces in *S. imperator* and *S. midas* to any of the habitat utilization and performance parameters, because both species never stood out in having similarly large values or frequencies in any of them. Rather, both covered shorter distances (Fig. 2) than *S. mystax* and *L. nigrifrons*, so it can be assumed that they did not have to exert higher forces than the species that covered longer distances. However, our morphological data imply that both these species experience substantial loads or, at least, can cope with such loads during take-off and landing. Perhaps, in comparison, the larger IMI in *L. nigrifrons* allow for an extended deceleration distance due to relatively elongated forelimbs, which is crucial to slow the body down when landing on the trunks [51]. *S. midas* and *S. imperator* may have compensated for this lack of elongation with relatively larger articular surfaces and more powerful muscles for shoulder stabilization [99]. Although these two species rarely landed on vertical rigid supports in our study, it should not be ignored that a rather rarely used ability may nevertheless be an ecologically significant activity and may have a major impact on musculoskeletal structures [100, 101]. Another explanation for the relatively more robust limb features in *S. midas* and *S. imperator* could be a possible increased risk of falls on thinner and more compliant branches which could result in a selection pressure towards more robust bones. It has been demonstrated that rare loading events, with high potential costs of failure, such as falls, will favor higher bone safety factors in comparison to habitual loads [see 102 for a review]. The reason for the falls could be various, such as breakage of the take-off or landing supports, or misjudgment of the distance and flexibility of the landing supports [103]. The falls we observed were always falls from horizontal or slightly oblique flexible supports. However, this only affected juveniles of *S. mystax*, which were not considered in this study. The frequency of free falls of *S. mystax* while foraging was three times higher than that of *L. nigrifrons* in a study by Peres [104]. And in a study by Price [103], *S. oedipus* was able to grasp lower branches and prevent falls to the ground only 30% of the time. Even from heights up to 10 m, tamarins were not observed to injure themselves by landing always on their legs [103]. This fact may suggest that *S. midas* and *S. imperator* have more robust bone features due to possible falls from compliant supports.

## Conclusion: weak integration of ‘levels’

Our study revealed that the three considered ‘levels’ of habitat utilization, leaping performance and locomotor morphology are surprisingly weakly integrated in our study system of tamarins. It also appears that specific variables of support use correspond to specific variables of leaping performance, while morphological differences cannot be linked meaningfully to any of these two ‘levels’ except for a few isolated variables. The intraspecific variability and the poor clustering on the two behavioral levels should caution against the assignment of species to coarse ecological categories (vertical leaper vs. horizontal leaper, or more general ones, e.g. fossorial vs. arboreal vs. cursorial etc.) for drawing ecomorphological inferences, as it is often done in large-scale interspecific studies, at any taxonomic level. Hypotheses on the form-function relationship depend on the degree of our previous understanding of the ecology of the animals, but this might be incomplete, offering much room for mis- and overinterpretation. Variations in the ecology of the animals and slight changes in behavior that were not considered in the respective study may influence and bias the inferences [2]. In our case, it may well be that the similar morphology of *S. mystax* and *L. nigrifrons* is determined by shared, but not leaping-related, behavioral characteristics [2] that were not considered in our study such as clinging to trunks during exudate feeding [105]. Also, sometimes similar morphologies may result from or respond to different selective pressures (i.e. ‘one-to-many mapping’ of form onto function [9, 106]; e.g. similar ulnar morphology may result from both digging and climbing in xenarthrans [107]). Additional behavioral data as well as studying other tamarin species might elucidate which aspects of support use and performance mainly drive the evolution of the locomotor apparatus. Our in-depth exploratory analysis, despite its practical limitations (e.g. two species being observed in a naturalist park instead in the wild; small interspecific sample size etc.), serves as a starting point for the generation of novel hypotheses.

### Supplementary Information

**Additional file 1: Figure S1. **Different types of trunk-to-trunk leaps in the studied tamarin species. **Figure S2. **Measurements of the humerus obtained in Geomagic. Cranial (A) and craniodorsal (B). **Figure S3. **Measurements of the ulna obtained in Geomagic. **Figure S4. **Measurements of the radius obtained in Geomagic. **Figure S5. **Measurements of the femur obtained in Geomagic. **Figure S6. **Measurements of the tibia obtained in Geomagic. **Figure S7. **Hierarchical trees of clustering methods. **Figure S8. **Scree plots for the dimensionality reduction analyses. **Figure S9. **Characterization of morphology clusters. The difference between Cluster 2 (C2) and Cluster 1 (C1) is illustrated on the scale of standard deviations for each variable. Standardization was also done to facilitate comparison among variables like it was done for principal component analysis. **Figure S10. **Boxplot for leaping distance of *L. nigrifrons*. **Supporting information note 1. **Additional information on quantification of internal bone structure. **Supporting information note 2. **Additional information on Body size correction of morphological data. **Supporting information note 3. **Additional information on Missing data imputation. **Supporting information note 4. **Additional information on sampling bias correction of performance data. **Supporting information note 5. **Additional information on inferential statistics.

**Additional file 2: Table S1.** Characteristics of trees within the seven Whittaker plots with the total number (N) and the percentage value of trees and categories for the score of height of stay of the monkeys during the focal animal scan method in Peru and “La vallée des singes”. **Table S2**. Percentages of utilized categories of the studied support characteristics and posture. **Table S3.** Specimens used for morphological analysis. **Table S4.** Effect of sample bias correction on sample sizes. **Table S5.** Numbers of observations. **Table S6.** Cluster validation indices. **Table S7.** Cluster characteristics, i.e., five most representative observations for each cluster within the levels of habitat utilization and leaping performance and posture. **Table S8.** Inferential statistics regarding the categorical behavioral variables. **Table S9. **Inferential statistics regarding the continuous behavioral variables.

## Data Availability

All data and R code necessary for the reproduction of the results are available on Figshare (10.6084/m9.figshare.24937131).
