# Supplementary material for: Linking morphology, performance, and habitat utilization: adaptation across biologically relevant ‘levels’ in tamarins
Source: BMC Ecol Evol. 2024 Feb 14;24:22. doi: 10.1186/s12862-023-02193-z (PMC10865561; doi:10.1186/s12862-023-02193-z)
Supplement: Supplementary file 1 — Additional file 1: Figure S1. Different types of trunk-to-trunk leaps in the studied tamarin species. Figure S2. Measurements of the humerus obtained in Geomagic. Cranial (A) and craniodorsal (B). Figure S3. Measurements of the ulna obtained in Geomagic. Figure S4. Measurements of the radius obtained in Geomagic. Figure S5. Measurements of the femur obtained in Geomagic. Figure S6. Measurements of the tibia obtained in Geomagic. Figure S7. Hierarchical trees of clustering methods. Figure S8. Scree plots for the dimensionality reduction analyses. Figure S9. Characterization of morphology clusters. The difference between Cluster 2 (C2) and Cluster 1 (C1) is illustrated on the scale of standard deviations for each variable. Standardization was also done to facilitate comparison among variables like it was done for principal component analysis. Figure S10. Boxplot for leaping distance of L. nigrifrons. Supporting information note 1. Additional information on quantification of internal bone structure. Supporting information note 2. Additional information on Body size correction of morphological data. Supporting information note 3. Additional information on Missing data imputation. Supporting information note 4. Additional information on sampling bias correction of performance data. Supporting information note 5. Additional information on inferential statistics. [file 12862_2023_2193_MOESM1_ESM.docx]

**Supporting information**


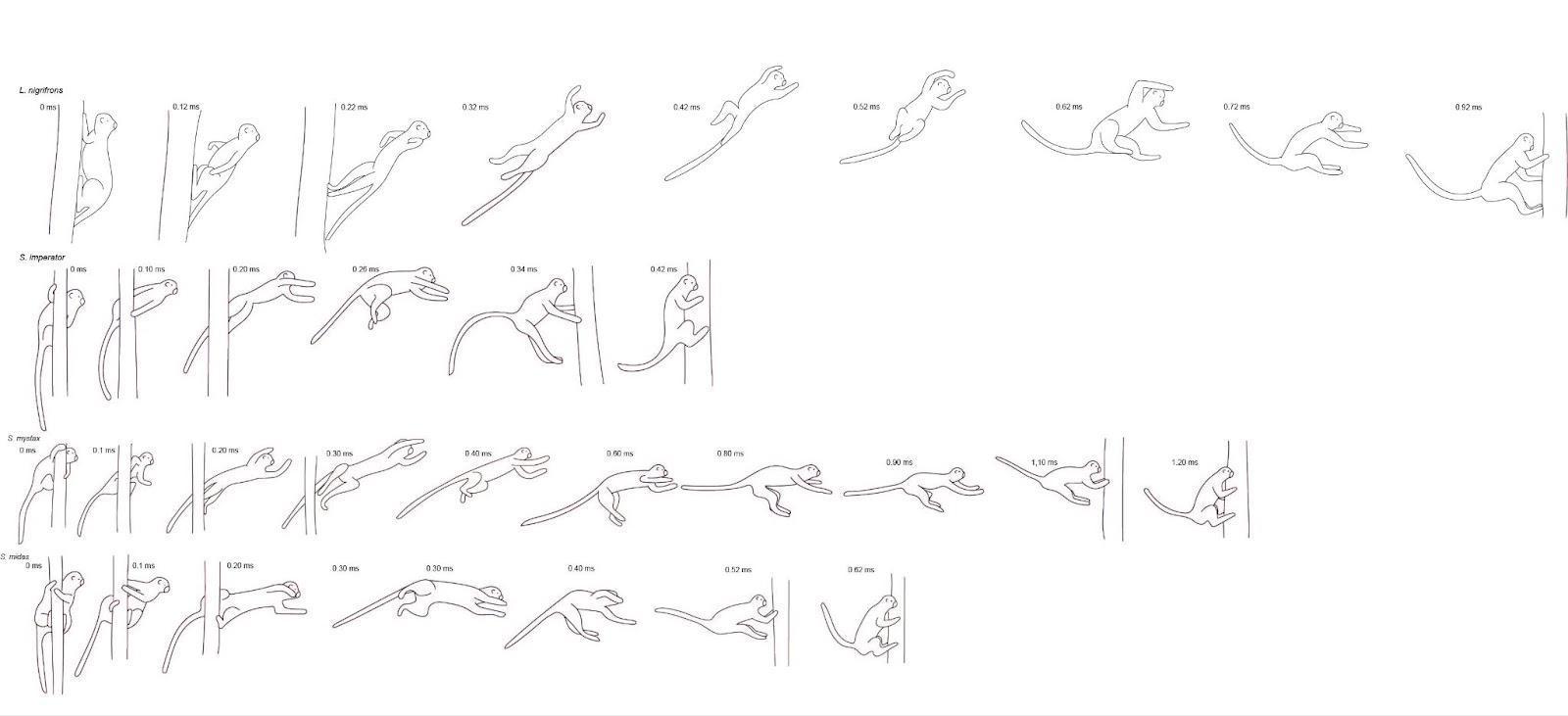


**Figure S1. Different types of trunk-to-trunk leaps in the studied tamarin species**


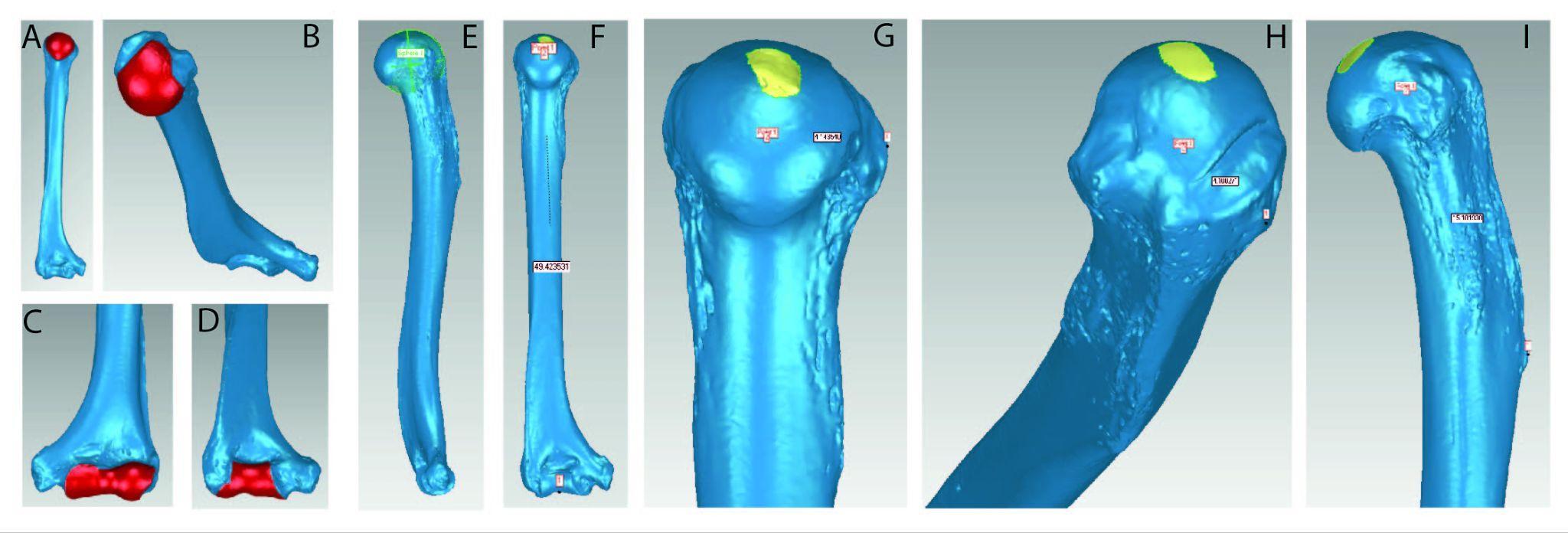


**Figure S2. Measurements of the humerus obtained in Geomagic.** Cranial (A) and craniodorsal (B) perspectives of the humeral head as well as cranial (C) and caudal (D) perspectives of the distal articulation sites with the two measured surface areas highlighted in red. A sphere with a center point was fit to the humeral head to approximate its center of rotation (E). The center of rotation was used to measure: the effective length of the humerus to the center of the trochlea (F), the in-lever of the subscapularis (G), the in-lever of the supraspinatus and infraspinatus (H), and the in-lever of the deltoideus (I).


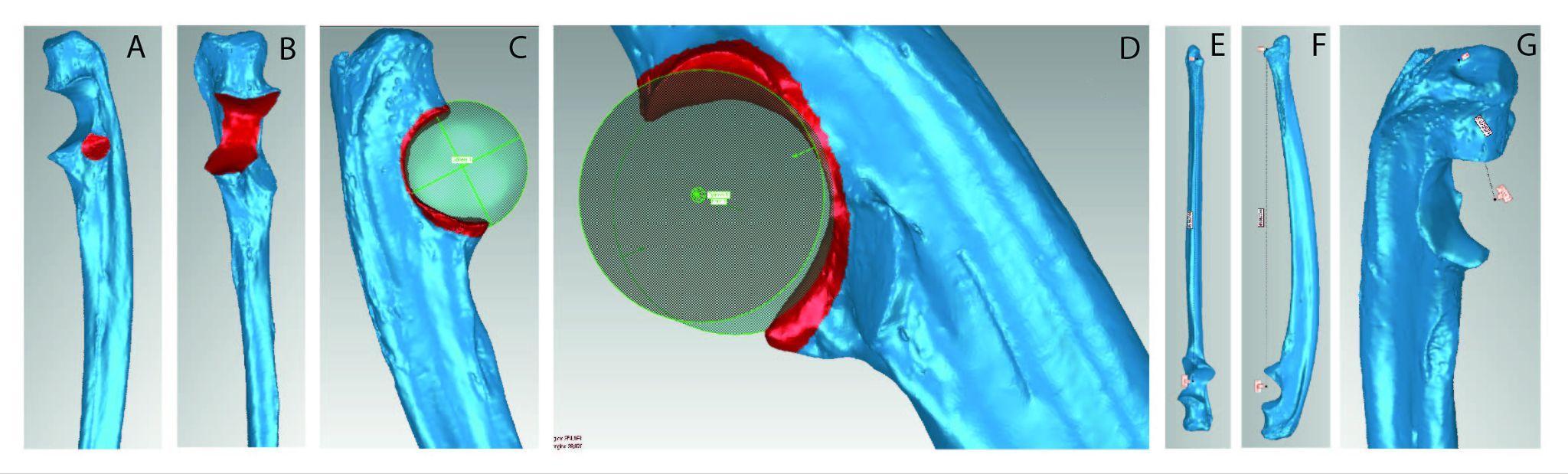


**Figure S3. Measurements of the ulna obtained in Geomagic.** Medial perspective of the radial notch (A) and cranial perspective of the semilunar notch (B) with the two measured surface areas highlighted in red. A sphere with a center point was fit to the semilunar notch to approximate its center of rotation (C) for obtaining length measurements. As the elbow joint is better modeled as a hinge joint, a cylinder would be better suited to estimate the axis of rotation. However, a fitted cylinder did not provide a single point that we could use to measure a distance to another point (see E-G). The sphere was used, because its center always felt close to the axis of rotation of the fitted cylinder (D) and because its mediolateral position was also considered appropriated for measuring the effective length of the ulna (E-F) and the in-lever of the triceps brachii (G).


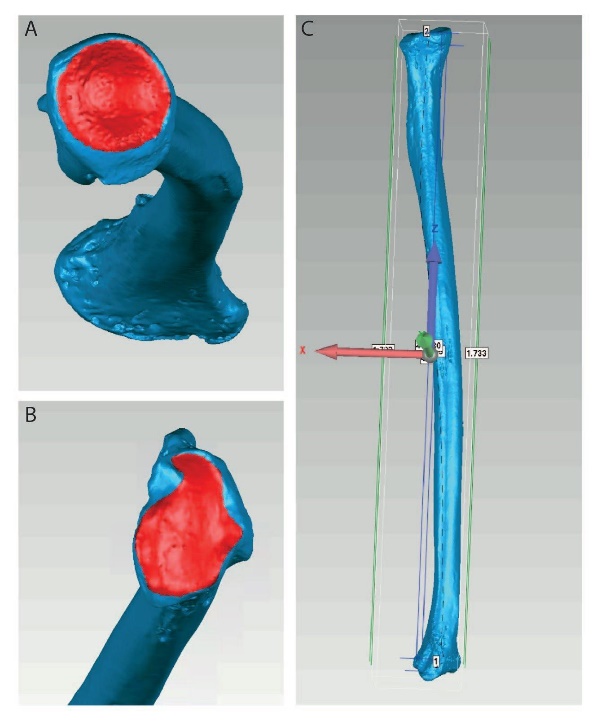


**Figure S4. Measurements of the radius obtained in Geomagic.** Proximal perspective of the articular notch of the radius head (A) and distal perspective of the articular surface to the os scaphoideum (B) with the two measured surface areas highlighted in red. Effective length of the radius (C).


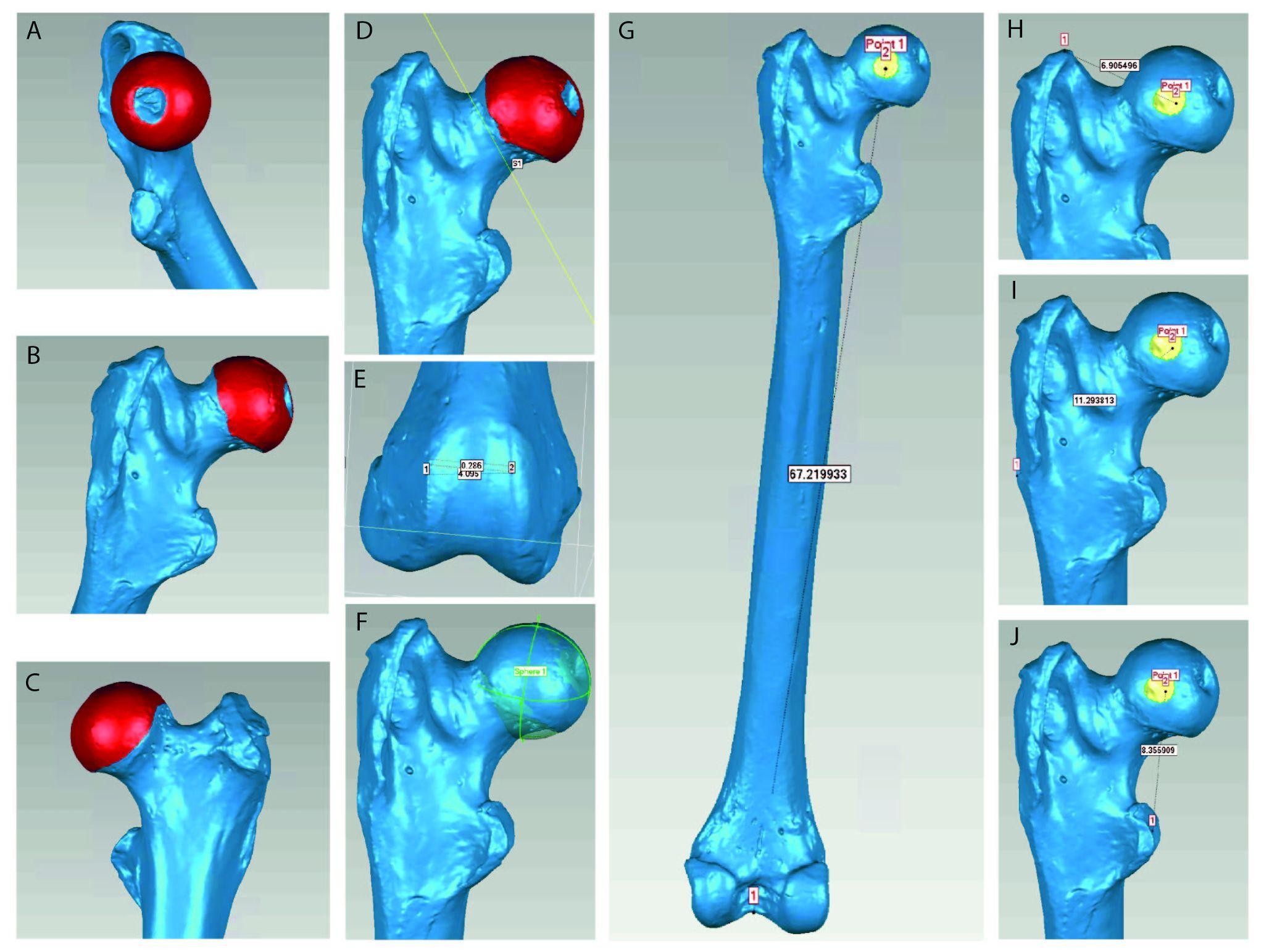


**Figure S5. Measurements of the femur obtained in Geomagic.** Caudal (A), ventral (B) and lateral (C) perspectives of the femoral head with the measured surface area highlighted in red. Diameter of the femoral neck (D). Measurement of the patella height (E). A sphere with a center point was fit to the femoral head to approximate its center of rotation (F). The center of rotation was used to measure: the effective length of the femur to the center of the condyli (G), the in-lever of M. gluteus medius (H), the in-lever of the M. gluteus superficialis (I), and the in-lever of the M. iliopsoas (J).


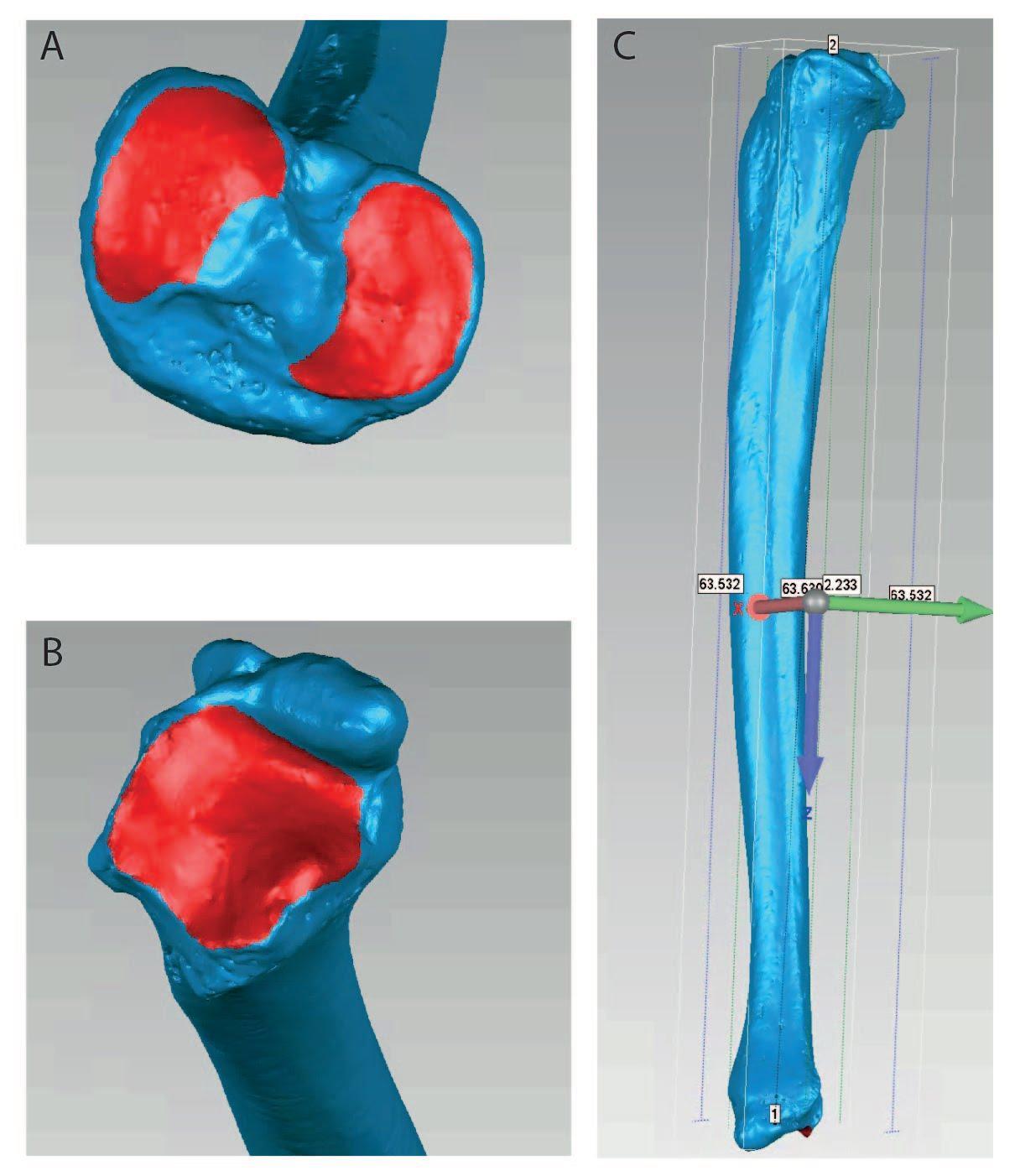


**Figure S6. Measurements of the tibia obtained in Geomagic.** Proximal perspective of the condyli notch (A) and distal perspective of the tibia-talus notch (B) with the two measured surface areas highlighted in red. Effective length of the tibia (C).


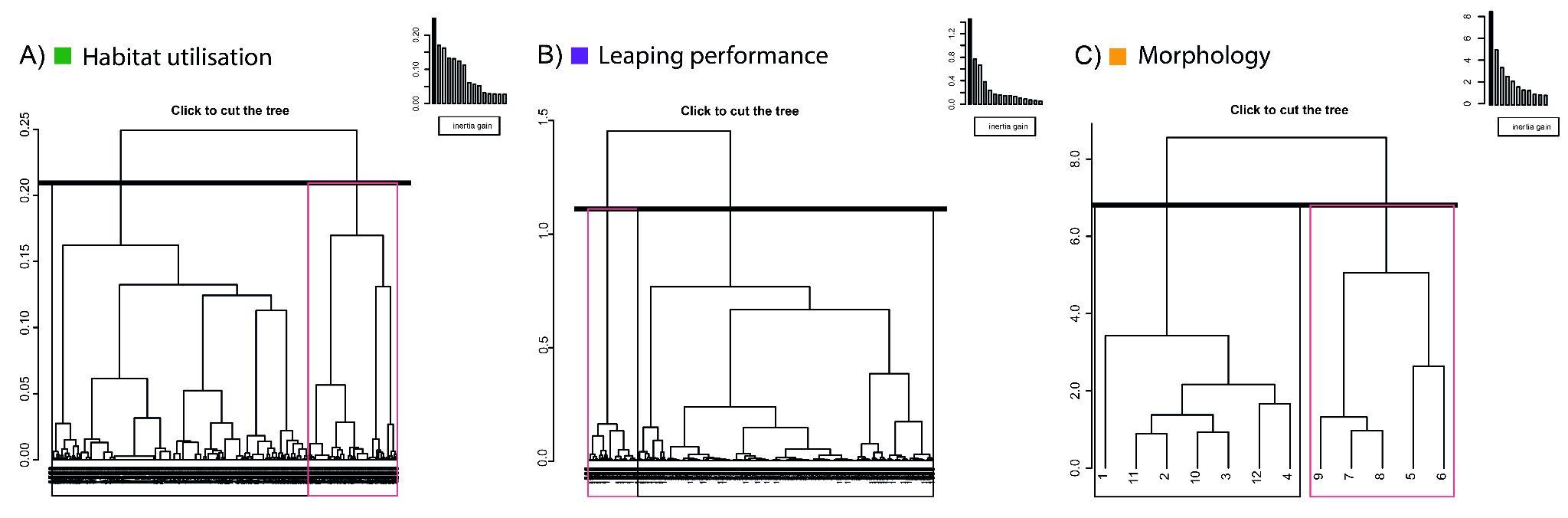


**Figure S7. Hierarchical trees of clustering methods**. Depicted are the chosen number of clusters based on graphical evaluation and inertia gain. The final assignments of observations to the clusters can be different due to the k-means consolidation conducted after hierarchical tree clustering. In case of the morphological dataset, for example, only three instead of five specimens were finally assigned to cluster 2. See Material and methods in the main document.


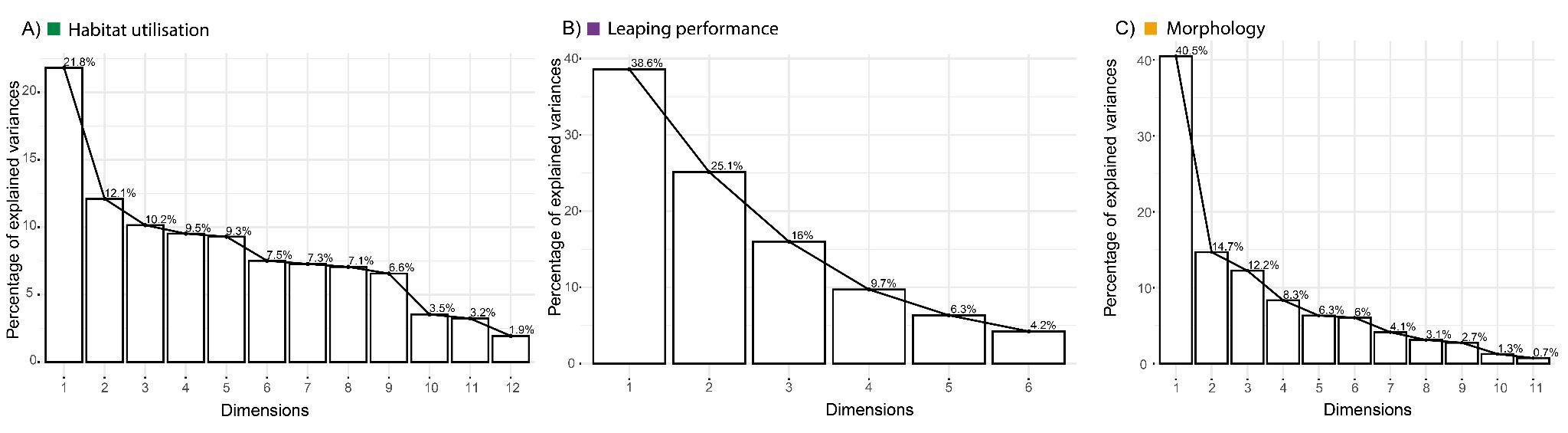


**Figure S8. Scree plots for the dimensionality reduction analyses**.

**
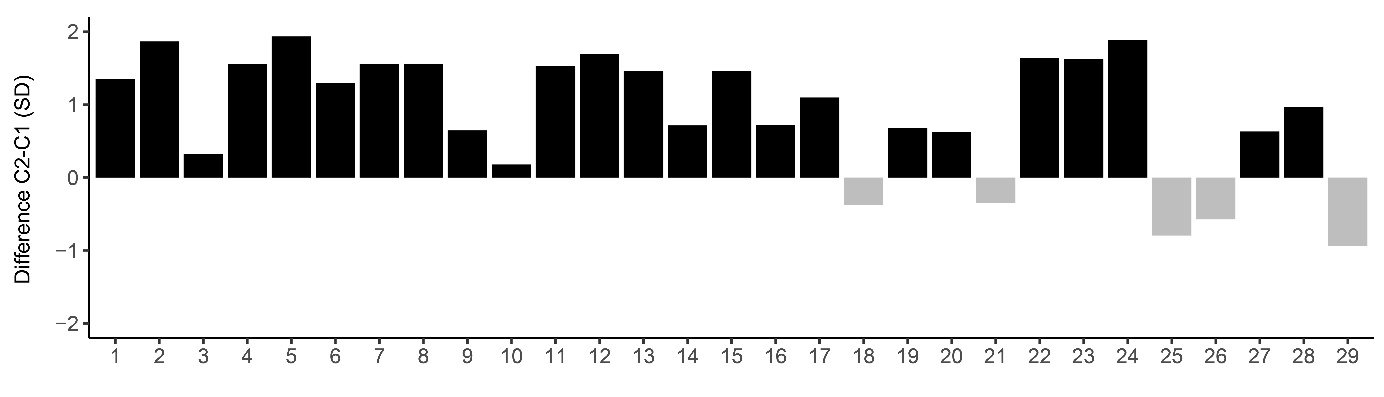
**

**Figure S9. Characterization of morphology clusters.** The difference between Cluster 2 (C2) and Cluster 1 (C1) is illustrated on the scale of standard deviations for each variable. Standardization was also done to facilitate comparison among variables like it was done for principal component analysis

**
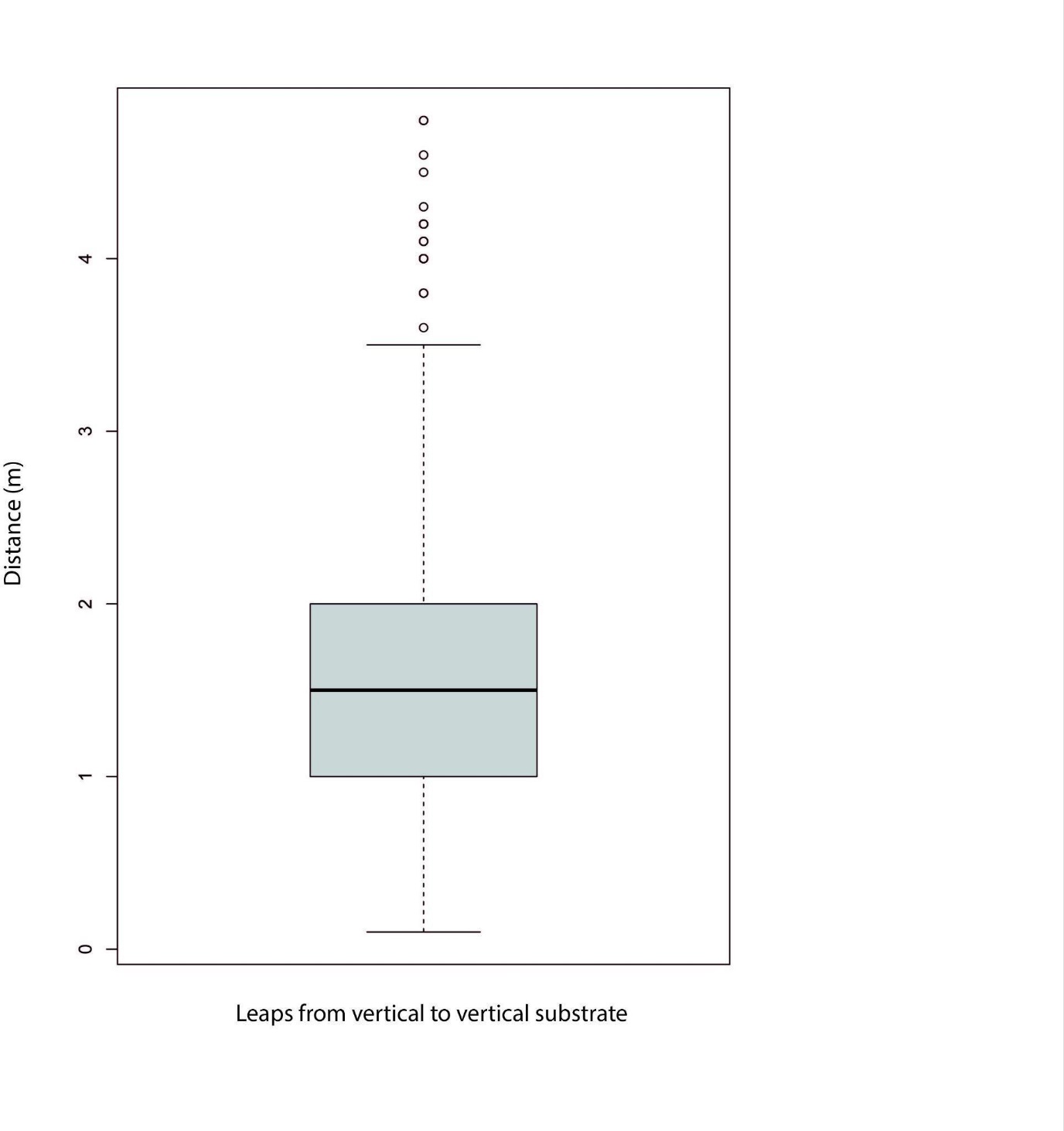
**

**Figure S10. Boxplot for leaping distance of *L. nigrifrons***.

**Supporting information note 1: Additional information on quantification of internal bone structure**

Among CSP we computed the cross-sectional area (CSA [mm^2^]) and the second moments of area along the mediolateral and anteroposterior directions (mlSMA [mm^4^] and apSMA [mm^4^]). While CSA is related to resistance to axial loadings, mlSMA and apSMA inform on bending loading regimes (Crowder and Stout 2011). CSA, mlSMA and apSMA were quantified in Fiji (Schindelin et al., 2012) on 2D cross-sections corresponding to the 50% level of the whole humeral and femoral lengths. This level has been considered the most informative in mammalian limb bones (Laurin, 2004) and used in CSP analyses of primate femur and humerus (e.g. Demes and Jungers 1993; Runestad 1994; Marchi et al. 2016). Once the 50% cross-section was isolated, it was thresholded (‘Optimise Threshold > Threshold Only’ routine) and purified (‘Purify’ routine). Then, after selecting the cross-section of interest (‘Wand (tracing)’ tool), CSA, mlSMA and apSMA were computed through the Fiji BoneJ plugin (‘Slice Geometry’ routine, Doube et al. 2010).

To characterize epiphyseal internal structure, the largest spherical region of interest (ROI) to only include cancellous bone was sampled from the humeral and the femoral head. The extraction of the largest ROI was chosen, instead of ROIs of fixed or scaled sizes (e.g. Ryan and Shaw 2012), to maximally represent trabecular architecture. The center of the sphere coincided with the center of the smallest rectangle encompassing the humeral/femoral head on the 2D section of the 50% of proximodistal length of the articular surface. See Alfieri et al. 2022 for details on ROIs extraction. The humeral and femoral head internal structure revealed non-complete epiphyseal fusion in two (*S. imperator* FMNH 98035 and *S. midas* FMNH 93236) and one specimen (*S. midas* FMNH 93236), respectively. Trabecular parameters, possibly biased in non-completely fused epiphyses, were not computed in these specimens. The diameters of the extracted ROIs range from 2.5 to 4 mm. On thresholded and purified (‘Optimise Threshold > Threshold Only’ and ‘Purify’ routines) ROIs, four trabecular parameters were extracted. Namely, we obtained the degree of anisotropy (DA, no unit, ‘Anisotropy’ BoneJ routine), informing on how much directionally stereotyped are the loadings on epiphyses, and the bone volume fraction (BV.TV., no unit, ‘Volume Fraction’ Bone J routine), proxy for the magnitude of stresses acting on joints (Kivell 2016). BV.TV., which by default is computed considering a cubic volume, was corrected accounting for a spherical volume (following Alfieri et al. 2022). Moreover, two additional parameters were computed to check the quality of the analyzed ROIs. The connectivity (Conn., no unit, ‘Connectivity’ BoneJ routine) represents a reliable proxy for the number of trabeculae included in the ROI. All ROIs exceeded the minimum accepted number of trabeculae (i.e. 50, Mielke et al. 2018). The trabecular thickness (Tb.Th., mm, ‘Thickness’ BoneJ routine) enables to assess the relative resolution of each ROI (i.e. Tb.Th/CT-scan resolution, Sode et al. 2008). The whole sample of ROIs exhibits a relative resolution going from 5.22 to 11.49 (average= 8.22), reaching or exceeding the minimum value suggested by Sode et al. 2008 and Kivell et al. 2011. After these checks, Conn. and Tb.Tb were excluded from subsequent analyses.

**Supporting information note 2: Additional information on Body size correction of morphological data**

Although tamarins are similar in body size, we decided to still account for small body size differences by normalizing the non-dimensionless morphological variables (the dimensionless variables were left as is). We used the sum of the centroid sizes of the humerus and the femur obtained from landmark data published by Botton-Divet and Nyakatura (2021) as a size proxy for each specimen (here simply referred to as CS). We divided each morphological variable by some power of the CS to obtain dimensionless variables (second moments of area divided by CS^4^, areas divided by CS^2^, lever-arms divided by CS).

**Supporting information note 3: Additional information on Missing data imputation**

For the performance dataset, only the three duration variables contained missing values. We first decided to keep only observations with at least one duration data point (1273 out of all 5920 leaps) and deleted two more cases with abnormally extended take-off durations, leaving 1271 cases for imputation, of which 92% had complete observations. We used the regularized iterative algorithm during factor analysis of mixed data (FAMD) implemented in the ‘imputeFAMD’ function to impute the missing values. FAMD was used since the performance level was characterized by continuous and categorical data. All behavioral variables were used and the number of significant dimensions (three) for imputation estimated via cross-validation as provided by the ‘estim_ncpFAMD’ function. Seven of the imputed values for flight and landing durations were smaller than the smallest observed value and sometimes even negative. These values were not considered meaningful and substituted by the smallest observed value of the original dataset of each respective variable.

Concerning the morphological dataset, missing values were present in the trabecular variables due to unfused epiphyses in two humeri and one femur. After body size correction of existing data, we used ‘estim_ncpPCA’ to obtain the significant numbers of dimension (one) and the ‘imputePCA’ function to impute the missing values.

**Supporting information note 4: Additional information on sampling bias correction of performance data**

To create a corrected dataset, we first merged the take-off and landing posture variables into a single variable with the four categories horizontal-horizontal, vertical-vertical, horizontal-vertical, vertical-horizontal (first term indicates take-off and second term landing posture). We used posture as a guideline, because it was the only performance variable available for all leaps. We used the following procedure to create an unbiased subsample of the performance dataset, which itself was a subsample of the habitat utilization dataset (Table S4). This procedure retained all leaps of the most underrepresented posture category and took random subsamples of leaps from the other categories (see Table S3 for absolute and relative frequencies before and after sample bias correction). First, we computed the relative frequencies of these four posture categories in the raw data set with all cases (Freqall, Table S3D) and in the subsampled performance dataset (Freqsub, Table S3E). We divided Freqall by Freqsub, so that values >1 indicated underrepresented categories and values <1 overrepresented categories in the subsample of each species. This showed, for example, a disproportionate abundance of vertical-vertical leaps, especially in *S. mystax*. In L. nigrifrons, on the other hand, the frequencies of horizontal-horizontal leaps were underrepresented (Table S3E). There were no or minor biases in the distributions of leaps in the two species filmed in the naturalistic park, *S. midas* and *S. imperator*. Nevertheless, we applied the correction to all four species with a minor effect on these two latter species. The four Freqall-to-Freqsub ratios were divided by their largest value (i.e., the most underrepresented category), yielding a new frequency distribution with the most underrepresented category having a value of 1 and the other three categories having a value below 1. The smaller the value, the stronger the category was represented in the subsample compared to the complete dataset. The absolute number of leaps per category in the subsample of a species (Table S3B) was then multiplied by its corresponding value in this frequency distribution. This gave us the number of leaps we had to sample from our performance dataset to match the relative frequency of posture categories in the leaps of the complete dataset. We used the R base function ‘sample’ to randomly draw leaps from the respective postural category of the behavioral dataset of each species. A seed was set before each sampling operation to ensure reproducible results. The sampling resulted in a selection of 1092 of the 1271 observations (Table S3C). Now, the relative frequencies of the posture categories after subsampling matched those of the complete dataset (compare Table S3F with Table S3D). The vertical-to-vertical leaps of *S. mystax* were affected the most with a selection of 19 out of 73 leaps to correct for overrepresentation (compare Tables S3C with Table S3A). Although this procedure resulted in the exclusion of much data for this category of this species, we deemed it indispensable to remove the bias towards a higher frequency of trunk-to-trunk leaps.

**Supporting information note 5: Additional information on inferential statistics**

For the categorical traits, we used χ²-tests with the Null-Hypothesis that there is no association between species and the respective trait’s categories. In case of a significant difference, we applied pairwise post hoc comparisons among all four species and corrected each p-value using the Bonferroni correction (i.e., multiplying the p-value by six with an upper p boundary of one). The R function ‘chisq.test’ was used to obtain these statistics.

For the continuous traits, we used the F-statistic of a linear regression model to test the null hypothesis that there is no mean difference between the four species. Since there were always significant differences in the variances between the four species according to a Levene test (Table S7, using the ‘Levene Test’ function in R), we used generalized least squares allowing for different error variances for each species. For this purpose, we used the ‘gls’ function in R. In case of a significant F-statistic, we post hoc conducted pairwise two-sided tests using the t-statistic from the ‘contrast’ function from the emmeans package (Lenth 2023). The p-values were corrected for multiple comparisons using the Bonferroni method as explained above. We used a significance level of 0.05 for all tests.

**References**

1. Alfieri, F., Botton-Divet, L., Nyakatura, J. A., & Amson, E. (2022). Integrative Approach Uncovers New Patterns of Ecomorphological Convergence in Slow Arboreal Xenarthrans. *Journal of Mammalian Evolution*, *29*(2), 283-312. https://doi.org/10.1007/s10914-021-09590-5
2. Botton-Divet, L.; Nyakatura, J. A. (2021). Vertical clinging and leaping induced evolutionary rate shifts in postcranial evolution of tamarins and marmosets (Primates, Callitrichidae). In: *BMC ecology and evolution* 21 (1), S. 132. https://doi.org/10.1186/s12862-021-01848-z
3. Crowder, C., & Stout, S. (Eds.). (2011). *Bone histology: an anthropological perspective*. CRC Press.
4. Demes, B., & Jungers, W. L. (1993). Long bone cross-sectional dimensions, locomotor adaptations and body size in prosimian primates. *Journal of human Evolution*, *25*(1), 57-74. https:// doi. org/ 10. 1006/ jhev. 1993. 1038
5. Doube, M., Kłosowski, M. M., Arganda-Carreras, I., Cordelières, F. P., Dougherty, R. P., Jackson, J. S., Schmid, B., Hutchinson, J. R. & Shefelbine, S. J. (2010). BoneJ: free and extensible bone image analysis in ImageJ. *Bone*, *47*(6), 1076-1079. https://doi.org/10.1016/j.bone.2010.08.023
6. Kivell, T. L. (2016). A review of trabecular bone functional adaptation: what have we learned from trabecular analyses in extant hominoids and what can we apply to fossils?. *Journal of Anatomy*, *228*(4), 569-594. https:// doi. org/ 10. 1111/ joa. 12446
7. Kivell, T. L., Skinner, M. M., Lazenby, R., & Hublin, J. J. (2011). Methodological considerations for analyzing trabecular architecture: an example from the primate hand. *Journal of anatomy*, *218*(2), 209-225. https:// doi. org/ 10.1111/j. 1469- 7580. 2010. 01314.x
8. Laurin, M. (2004). The evolution of body size, Cope's rule and the origin of amniotes. *Systematic Biology*, *53*(4), 594-622. https:// doi. org/ 10. 1080/10635 15049 04457 06
9. Lenth R (2023). _emmeans: Estimated Marginal Means, aka Least-Squares Means_. R package version 1.8.6
10. Marchi, D., Ruff, C. B., Capobianco, A., Rafferty, K. L., Habib, M. B., & Patel, B. A. (2016). The locomotion of Babakotia radofilai inferred from epiphyseal and diaphyseal morphology of the humerus and femur. *Journal of Morphology*, *277*(9), 1199-1218. https:// doi. org/ 10. 1002/ jmor. 20569
11. Mielke, M., Wölfer, J., Arnold, P., van Heteren, A. H., Amson, E., & Nyakatura, J. A. (2018). Trabecular architecture in the sciuromorph femoral head: allometry and functional adaptation. *Zoological letters*, *4*(1), 1-11. https://doi.org/10.1186/s40851-018-0093-z
12. Runestad, J. A. (1994). Humeral and femoral diaphyseal cross-sectional geometry and articular dimensions in prosimii and platyrrhini (primates) with application for reconstruction of body mass and locomotor behavior in adapidae (primates: eocene). The Johns Hopkins University.
13. Ryan, T. M., & Shaw, C. N. (2012). Unique suites of trabecular bone features characterize locomotor behavior in human and non-human anthropoid primates. *PloS one*, *7*(7), e41037. https:// doi. org/ 10.1371/ journ al. pone. 00410 37
14. Schindelin, J., Arganda-Carreras, I., Frise, E., Kaynig, V., Longair, M., Pietzsch, T., ... & Cardona, A. (2012). Fiji: an open-source platform for biological-image analysis. *Nature methods*, *9*(7), 676-682. https://doi.org/10.1038/nmeth.2019
15. Sode, M., Burghardt, A. J., Nissenson, R. A., & Majumdar, S. (2008). Resolution dependence of the non-metric trabecular structure indices. *Bone*, *42*(4), 728-736. https:// doi. org/ 10. 1016/j. bone. 2007. 12. 004
